# A systematic review of the effectiveness of participatory, health system-based interventions to improve the sexual and reproductive health and rights of adolescent girls and young women in Sub-Saharan Africa

**DOI:** 10.1080/26410397.2026.2643037

**Published:** 2026-03-18

**Authors:** Carol Vlassoff, Fahimeh Abdollahi, Peter Farrell, Lauren Davies, Prithi Ravichandran, Rachel Strohm, Chloe Champion, Kazeem Arogundade, Alison Krentel

**Affiliations:** a Adjunct Professor, School of Epidemiology and Public Health, Faculty of Medicine, University of Ottawa, Ottawa, Canada.; b MPH Candidate, School of Epidemiology and Public Health, Faculty of Medicine, University of Ottawa, Ottawa, Canada; c Research Librarian, Library, University of Ottawa, Ottawa, Canada; d Summer Student, Threads Lab, Bruyère Health Research Institute, Ottawa, Canada; e Intern, Threads Lab, Bruyère Health Research Institute, Ottawa, Canada; f Community of Practice Manager, Threads Lab, Bruyère Health Research Institute, Ottawa, Canada; g Global Health Practicum Student, Threads Lab, Bruyère Health Research Institute, Ottawa, Canada; h Consultant, FAST Package, Threads Lab, Bruyère Health Research Institute, Ottawa, Canada; i Associate Professor, School of Epidemiology and Public Health, Faculty of Medicine, University of Ottawa, Ottawa, Canada; Principal Investigator, Threads Lab, Bruyère Health Research Institute, Ottawa, Canada

**Keywords:** adolescents, girls, sexual and reproductive health and rights, interventions, participatory, health system, Sub-Saharan Africa, young women

## Abstract

This systematic review investigates the impact of participatory health-sector led interventions on female adolescent sexual and reproductive health and rights (ASRHR) in Sub-Saharan Africa. Adolescents face many challenges, including high rates of HIV and other risk factors. Interventions to promote ASRHR are therefore critical for enhancing their overall wellbeing. Our peer-reviewed search yielded 6225 articles from online databases, which we imported to Covidence. 2619 duplicates were removed, leaving 3606 articles that two authors screened by title and abstract. 3545 articles not meeting our inclusion criteria were removed, and 61 full-text articles were screened, also by two authors. Only four articles met our eligibility criteria. The interventions in the selected studies included HIV testing preferences in Zambia, layered interventions in Malawi, peer support for HIV testing and adherence in Uganda, and a participatory curriculum in Zimbabwe. Important results included the value adolescents attached to interventions delivered by health providers; the need for interventions to address ASRHR issues in a comprehensive way; and the need for more rigorous indicators of the nature and role of peer support in ASRHR interventions. Several unexpected findings included the paucity of studies on participatory youth-friendly interventions delivered by the health sector, the dominance of adolescent research on HIV issues and the neglect of other priorities, and the limited research attention to adolescent rights. We conclude that investing in the formation and sensitisation of African health workers to adolescent needs can have a positive and sustainable impact, although further research is needed to validate these findings.

## Plain language summary

This study looks at the problems faced by teenage girls in Africa when it comes to their sexual and reproductive health and rights, and what is being done to help them. One of the big problems they face is the fear of getting infected by HIV, preventing many of them from reaching their goals and living full, healthy lives. In this paper, we describe a review of programs meant to improve the sexual and reproductive health and rights of teenagers in Africa. We focus on those provided by health services, and that also involve teens in the planning and program activities. We reviewed over 3600 studies on the topic. However, only four studies fit all of our requirements. Mostly, this was because most programs were not provided by health services or did not involve the girls themselves in their planning and decision-making. The studies we found included: (1) a program in Malawi that used different educational strategies to inform girls; (2) a peer support group in Uganda to help girls get tested for HIV and stick with treatment; (3) a school program in Zambia for youth already infected with HIV; and (4) a study in Zimbabwe on what types of HIV testing girls preferred. All the studies found that girls worried about getting HIV or another disease through having sex. However, they also wanted help with other problems related to being female, such as being treated unfairly at home and being expected to do household chores while boys were not. Girls preferred to go to health facilities for help with these problems because they felt health workers were knowledgeable. They wanted health services to be welcoming and open to listening to them when they talked about their concerns. They also liked it when other girls their age were trained about these issues and could answer personal questions one-on-one. They felt that programs meant for young people should include them in the planning and program activities and that they should be encouraged to give feedback when they wanted to do so.

## Résumé

Cet examen systématique étudie l’impact des interventions participatives menées par le secteur de la santé en matière de santé et de droits sexuels et reproductifs des adolescentes en Afrique subsaharienne. Les adolescentes sont confrontées à de nombreuses difficultés, notamment des taux élevés de VIH et d’autres facteurs de risque. Les interventions destinées à promouvoir la santé et les droits sexuels et reproductifs sont donc essentielles pour améliorer leur bien-être général. Notre recherche, évaluée par des pairs, a permis d’identifier 6225 articles dans des bases de données en ligne, articles que nous avons importés dans Covidence. Après suppression de 2619 doublons, nous avons gardé 3606 articles dont le titre et le résumé ont été examinés par deux auteurs. 3545 articles ne répondant pas à nos critères d’inclusion ont été exclus et 61 articles en texte intégral ont été examinés, également par deux auteurs. Seuls quatre articles répondaient à nos critères d’éligibilité. Les interventions des études sélectionnées incluaient les préférences en matière de dépistage du VIH en Zambie, des interventions à plusieurs niveaux au Malawi, le soutien par les pairs pour le dépistage du VIH et l’observance du traitement en Ouganda, et un programme d’enseignement participatif au Zimbabwe. Parmi les résultats importants figuraient l’intérêt que les adolescentes accordent aux interventions assurées par les prestataires de santé; la nécessité d’interventions qui abordent les questions de santé et de droits sexuels et reproductifs de manière globale; et le besoin d’indicateurs plus rigoureux de la nature et du rôle du soutien par les pairs dans les interventions en matière de santé et de droits sexuels et reproductifs. L’examen a obtenu plusieurs résultats inattendus: la rareté des études sur les interventions participatives adaptées aux jeunes mises en œuvre par le secteur de la santé; la prédominance du VIH dans les recherche sur les adolescents et le manque d’intérêt pour d’autres priorités; et le peu d’attention accordée aux droits des adolescents dans la recherche. Nous concluons que des investissements en faveur de la formation et la sensibilisation des professionnels de santé africains aux besoins des adolescents peuvent avoir un effet positif et durable, même si des recherches supplémentaires sont nécessaires pour confirmer ces résultats.

## Resumen

Esta revisión sistemática investiga el impacto de intervenciones participativas lideradas por el sector salud sobre la salud y los derechos sexuales y reproductivos de las adolescentes (SDSRA) en África subsahariana. Las adolescentes enfrentan muchos retos, tales como altas tasas de VIH y otros factores de riesgo. Por ello, las intervenciones que promueven SDSRA son fundamentales para mejorar su bienestar general. Nuestra búsqueda revisada por pares produjo 6225 artículos de bases de datos digitales que importamos a *Covidence*. Tras eliminar 2619 duplicados, quedaron 3606 artículos que dos autores examinaron por título y resúmenes. Se eliminaron 3545 artículos que no reunían nuestros criterios de inclusión, y los dos autores examinaron el texto completo de 61 artículos. Solo cuatro artículos reunían nuestros criterios de elegibilidad. Las intervenciones en los estudios seleccionados abarcaron preferencias de pruebas de VIH en Zambia, intervenciones escalonadas en Malaui, apoyo de pares para pruebas de VIH y adherencia en Uganda, y un plan de estudios participativo en Zimbabue. Los resultados importantes fueron el valor que las adolescentes atribuyeron a las intervenciones realizadas por prestadores de servicios de salud; la necesidad de que las intervenciones trataran los asuntos de SDSRA de una manera integral; y la necesidad de tener indicadores más rigurosos de la naturaleza y función del apoyo de pares en las intervenciones de SDSRA. Los hallazgos inesperados fueron la escasez de estudios sobre intervenciones participativas adaptadas a jóvenes y realizadas por el sector salud; el predominio de la investigación adolescente en asuntos relacionados con el VIH y el descuido de otras prioridades; y el hecho de que las investigaciones prestaran atención limitada a los derechos de las adolescentes. Concluimos que invertir en la formación y sensibilización del personal de salud africano con relación a las necesidades de las adolescentes puede tener un impacto positivo y sostenible, aunque es necesario realizar más investigaciones para validar estos hallazgos.

## Introduction

There are 1.3 billion adolescents (defined as youth aged 10–19 years), in the world today, constituting 16% of the global population, more than ever before.^1^ Adolescents comprised about 22% of Africa’s estimated population in 2024,^2^ nearly one-fifth of adolescents worldwide.^2^ In 2023, there were an estimated 641 million adolescent girls globally, and 145 million adolescent girls living in Africa. The Global Strategy for Women’s, Children’s and Adolescents’ Health (2016–2030), released by the United Nations Secretary General in 2015, envisions, by 2030,

“*a world in which every woman, child and adolescent in every setting realizes their rights to physical and mental health and wellbeing [including sexual and reproductive health and rights], has social and economic opportunities, and is able to participate fully in shaping prosperous and sustainable societies*”.^3^

Adolescent sexual and reproductive health and rights (ASRHR) is a broad term encompassing freedom from

“*coercion and intimate partner violence; lack of education and information; high rates of early and unwanted pregnancy; lack of access to health services, especially for contraception and safe abortion; gender inequalities and harmful traditional practices, such as female genital mutilation (FGM) and child, early and forced marriage; and risk of STIs (including HIV)*”.^4^

Yet too many women, adolescent girls, and children lack access to basic human amenities, such as quality healthcare and education, clean air, water, sanitation, and adequate nutrition.^3^ ASRHR are restricted by obstacles such as HIV, sexually transmitted infections (STIs), early pregnancy and childbearing, violence, female genital mutilation, and female genital schistosomiasis, among others, inhibiting them from reaching their full potential. These barriers are especially prevalent among young women and girls in Africa, largely due to its high rates of HIV. In 2021, 59% of new HIV infections globally were in the African continent, and 63% of new infections occurred among adolescent girls and young women (AGYW).^5^ Adolescent pregnancy was 91 per 1000 AGYW in 2023 compared to 41.3 per thousand globally.^6^ While these rates are declining globally, including in Africa, progress has been slowest in Sub-Saharan Africa (SSA). Other indicators of adolescent health and wellbeing are also low: for example, the lifetime prevalence of intimate partner violence against girls aged 15–19 was as high as 40% in Central SSA compared to 24% globally.^7^ Although SSA comprised only 15% of the global population in 2022, it experienced 56% of child and adolescent deaths in 2021, and 72% of maternal deaths in 2020.^8^ Anaemia among children and adolescents remains high, especially among pregnant adolescents.^9^ In 2018, its schooling rates were much lower than all other regions: only 40% of girls in SSA completed secondary education compared to 75% of girls worldwide.^10^ Each year, more than 4 million girls globally are at risk for female genital mutilation (FGM), with the majority being cut before the age of 15,^11^ and it is estimated that approximately 20 million adolescent girls aged 12–19 are affected by female genital schistosomiasis (FGS) in SSA.^12^

Measuring progress toward the achievement of the Global Strategy targets is limited by a lack of standardised tools to assess the health, nutrition and well-being of adolescents in SSA.^13^ However, available data suggest that progress has been slower than anticipated. A recent Lancet review noted that 48 countries in SSA continue to have the world’s highest fertility rates, poorest health indicators, weakest health systems, and difficult socioeconomic conditions.^8^

The failure of African countries to maintain the momentum toward their expected adolescent health attainments can be explained by a “polycrisis” of daunting global and regional challenges, including the protracted consequences of COVID-19 and other recurring disease outbreaks, armed conflict, climate change, economic and political uncertainties, and growing public debt. Furthermore, recent cutbacks in international development assistance (in the order of 70% between 2021 and 2025)^14^ have created unprecedented shortfalls in the capacity of Africa’s already fragile health systems to furnish essential services and programs for women, children and adolescents.

The health sectors globally and regionally are the principal duty bearers responsible for providing available, accessible, acceptable, and quality health (AAAQ) services to their citizens in a nondiscriminatory manner under the Right to Health in WHO’s Constitution of 1946 and the Declaration of Human Rights of 1948. Reciprocally, the rights holders, including adolescents, are responsible for knowing and claiming their freedoms and entitlements – namely, the right to control one’s health, informed consent, bodily integrity, and participation in health-related decision-making, freedom from torture, mistreatment, and harmful practices.^15^ Human rights principles, including participation, accountability, equality, and non-discrimination, should be an integral part of all stages of health programming. As health systems and services in Africa increasingly confront the challenges of self-reliance and efficient ways of meeting them, evidence of successful interventions, in line with their human rights obligations and principles, can help pave the way for adolescent health approaches and services that are welcoming, effective, equitable, and sustainable. Positive interventions to promote adolescents’ sexual and reproductive health and rights are therefore critical for enhancing their physical, emotional, mental, developmental, social, and economic wellbeing.^16^

In this review, we prioritised research on interventions delivered primarily through the health sector for several reasons. First, interventions or programs integrated into existing health services are likely to be more sustainable than those provided by other sectors or non-governmental organisations (NGOs). Second, the health system is responsible for providing SRHR services to adolescents, it is the duty bearer of their health-related human rights,^17^ and it is where most adolescents ultimately need to access care. Third, barriers to the use of the formal health services by African adolescents have been widely documented, including fear of receiving reprimands or judgmental attitudes from health workers, anticipated stigmatisation from community members, and/or lack of access to youth-friendly health services (YFHS).^18–24^ Focusing on interventions delivered by the health sector, despite these barriers, provides an opportunity to better understand and overcome them. Fourth, there is limited documentation about constraints faced by health services themselves in providing YFHS to their clients. Therefore, we were interested in interventions within the health services that addressed such barriers and their effectiveness in achieving their desired results. We were primarily interested in assessing the impact of the interventions, while feasibility and acceptability were a secondary objective.

In conceptualising this review, we were influenced by the concept of participatory approaches as expounded by Paulo Freire, a Brazilian educator and philosopher, in which those for whom the interventions are intended are active participants in their development, not merely recipients.^25^ Freire’s philosophy maintains that engaging the intended beneficiaries of the intervention in identifying challenges and potential solutions inspires a critical consciousness and self-awareness that helps people solve their own problems. Freire encouraged dialogue and co-learning between the facilitators of an intervention (a more privileged teacher or researcher) and its recipients (students, study subjects, “the oppressed”); a transformative, continuous cycle of joint reflection and action. His approach has inspired generations of educators and social change activists worldwide and has been applied, though less frequently, in the public health domain.^26–30^ Closely aligned with Freire’s philosophy are the principles of human rights which recognise the dignity of every human being, their right to challenge oppression, and to strive for social change. While we did not anticipate or require that our selected studies explicitly recognise Freire’s principles, we did require that their participatory nature align with his transformative philosophy and methods. We return to this alignment in the Discussion section below.

The potential of participatory interventions for enhancing healthy behaviour among African youth (both males and females) has been demonstrated in several studies of interventions conducted outside the health sector. These include participatory approaches with refugee youth through comic book scenarios highlighting sexual health issues^31^; a “designathon” consisting of youth-initiated suggestions to improve linkage to care and sexually transmitted infections (STI) services in Nigeria^32^; engaging youth in designing an SRH intervention in Zambia^33^; a national digital health platform to improve reproductive health among Rwandan youth^34^; understanding barriers to voluntary counselling and testing (VCT) for HIV in Uganda^35^; co-designing preferred content of adolescent health check-ups in Zimbabwe^36^; and adolescents’ portrayal of an ideal health clinic in South Africa.^37^ We sought to identify similar or additional participatory interventions within SSA health services.

The objective of this systematic review is therefore to determine the effectiveness of participatory interventions, delivered through the health services, in improving the sexual and reproductive health and rights of adolescent girls and young women aged 10–19 years in Africa, a priority issue for our research team. Existing examples of successful interventions provide potentially replicable strategies for application and scaling up in different African settings with different ASRHR challenges.

## Methods

**Figure 1.**
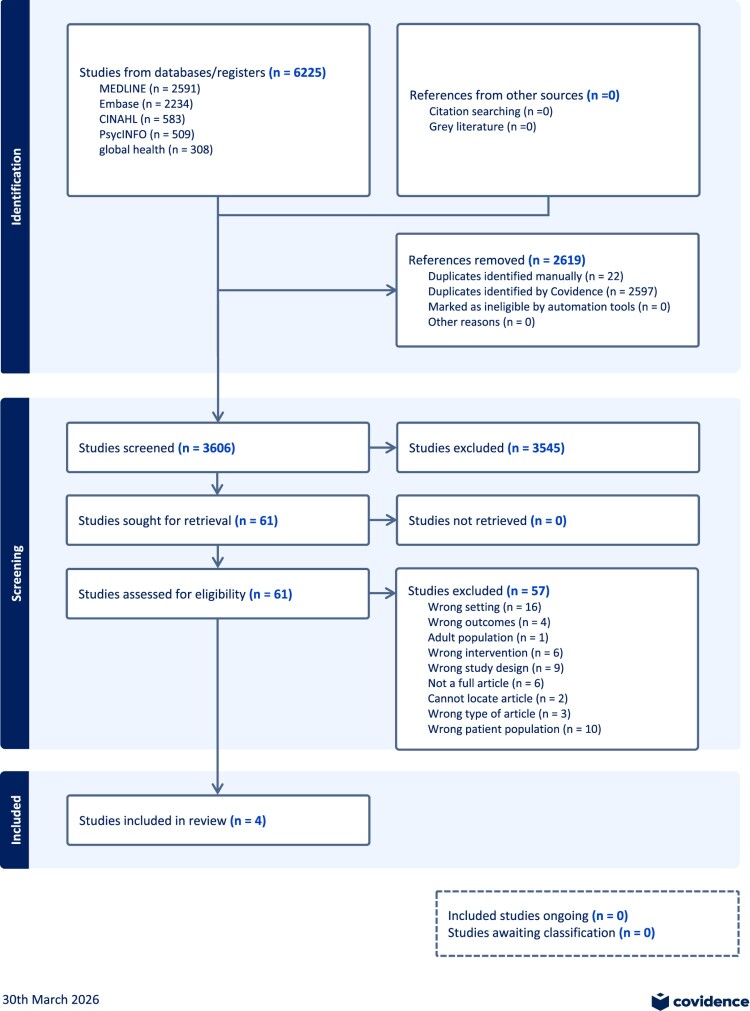
PRISMA flowchart

This systematic review is in adherence with the Preferred Reporting Items for Systematic Reviews and Meta-Analysis (Figure 1).^38^ The protocol was registered with the PROSPERO (CRD42023446937). As the review is based entirely on secondary data, no ethical approval from the authors’ institutions was required.

### Eligibility criteria

Included studies were limited to peer-reviewed primary studies, reported in English. No date restrictions were applied. We used a PICO framework to guide the development of our search strategy:

- Population: Adolescent girls and young women (aged 10–19 years) living in sub-Saharan Africa. We included articles that exceeded this age group (e.g. females aged 10–24) providing they reported on females aged 10–19, or a portion of this age group, separately. Similarly, we included studies with males, providing that they reported on our female focus population separately.
- Interventions: ASRHR interventions using participatory methods delivered through the health sector but can be combined with other sectors. Interventions of interest could be programs, services, or projects. The participatory nature of the intervention was assessed by determining if it involved input from adolescent girls in one or more key activities of the intervention, not just as respondents to interviews or post-project evaluations. Key activities included co-design, free selection of project activities by participants who carried them out independently (without specific instructions from project implementers), youth involvement in proposing problems and solutions, or evaluation of project activities about which youth were aware from the start of the intervention, or a combination of these interventions.
- Comparator/Control: Preferred if available but not required.
- Outcome: Must be clearly stated. Either quantitative or qualitative, or both.

All articles resulting from the search were screened through Covidence using inclusion and exclusion criteria listed in Table 1. We considered Sub-Saharan Africa those 48 countries located in the African Union’s South, East, and West sections (listed in the ASRHR Supplemental File Search Strategies). We considered an intervention related to ASHRH if it was one of the following topics: sexual health education or promotion; family planning and contraception including abortion; gender-based violence; and prevention, diagnosis, and treatment of STIs, including HIV.

**Table 1.** Inclusion and exclusion criteria for systematic review

Inclusion criteria	Exclusion criteria
Setting: Study conducted in Sub-Saharan Africa	Study conducted outside Sub-Saharan Africa
Study population: Girls aged 10–19	Other study population
Study methods: Qualitative, quantitative (randomised controlled trials; before/after comparison; cross-sectional comparisons); Mixed	Any other study methods; systematic reviews
Intervention: Must be delivered through/led by health sector Must be participatory, involving input of adolescent girls into one or more key implementation activities	Interventions not delivered through health sector. Intervention is not participatory or involves adolescent girls only after completion, such as in the post-intervention evaluation
Outcomes: Effectiveness in motivating change, such as use of ASRHR services or clinical outcomes, assessed	Studies focused on other issues, and there was no assessment of the change produced by the intervention

### Search strategy

A sensitive multi-database search strategy was designed by one of the co-authors, a health sciences librarian (PF), and then peer-reviewed by a second librarian using the Peer Review of Electronic Search Strategies tools,^39^ and was deposited in Open Science Framework (OSF) (https://osf.io/9a6qc; also available as supplemental information). The following information sources were searched to provide broad coverage of health sciences and global health literature. All relevant literature in MEDLINE (Ovid), Embase (Ovid), PsycInfo (Ovid), CINAHL (EBSCOhost), and Global Health (EBSCOhost) were included up to October 27, 2023. Results were limited to reports published in English. The search consisted of five concepts: sexual health and reproductive health rights topics; adolescents; Sub-Saharan Africa; youth involvement; and health interventions. The strategy was informed by previously conducted systematic searches.^40,41^ Search results were exported to Covidence and duplicates were eliminated using the platform’s duplicate identification feature. To supplement the database search, the context articles identified during screening (described in the next section) were scanned for mention of relevant studies.

### Article screening, data extraction, and data analysis

For the Covidence screening, two (of four) authors (LD, PR, RS, CC) screened the title and abstracts of the initial studies to remove duplicates and those not meeting our inclusion criteria, with an additional author resolving conflicts (CV, AK). Studies meeting our inclusion criteria were moved to full text screening, which was conducted by two (of four) authors (CV, LD, PR, RS), each article screened by two of them, with another author (AK) resolving conflicts. The authors involved in the screening met weekly to review progress and discuss any concerns.

*Thematic analysis* was conducted by jointly selecting the main themes or categories of investigation in the eligible studies, and then searching each article for information. Data from the selected eligible articles were extracted using a Covidence template that was modified by the authors according to our study criteria, summarised in Table 2 and detailed in Supplemental Table 1. Our data analysis involved three main steps: quality assessment, thematic analysis, and ranking of intervention effectiveness, conducted by four authors (FA, PR, CV, LD).

**Table 2.** Salient characteristics of included studies (See Supplementary Table 1 for complete data extraction details)

Reference	Country	Study subjects	Intervention	Degree of participation	Health sector involvement	Intervention effectiveness
Nakalega et al, 2023	Uganda	30 females aged 18–24	Peer-delivered HIV test kits, preventive medication (PrEP)*, counselling, and ART** adherence support provided regularly	Ongoing interactions with peers and scheduled clinic visits	*High* – Nurses are involved in the provision of supplies to peers and the testing of results	High acceptance of peer-delivered services and support, especially among AGYW*** engaged in sex work, facilitated by regular education and counselling
Stangl et al, 2021	Zambia	21 females aged 15–19	Curriculum workshop with HIV + AGYW, including activities supporting HIV disclosure, ART adherence, stigma reduction, hope for the future, grief and loss	High participation of AGYW in attending a minimum of 5 out of 6 sessions	*Low* – Intervention co-designed with healthcare providers who also selected and screened study participants	Positive feedback from participants on co-facilitation by HIV+ peers and adult counsellors. Qualitative data supported a positive impact on ART adherence and hope for the future No significant quantitative impact on ART adherence, hope for the future, or stigma reduction
Mavodza et al, 2021	Zimbabwe	700 females aged 16–24 (quantitative data) 26 females aged 16–19 (qualitative data)	Three options for HIV testing are offered: by provider; HIV self-testing on-site, no provider; off-site alone	Participation through consultations with health staff and focus group and in-depth interviews feedback from participants	*High* – Health workers at primary care clinics performed testing	Youth preferred provider testing at a trusted youth-friendly service Hesitancy regarding self-testing Finds need for greater investment in youth-friendly service provision
Manda et al, 2021	Malawi	31 females aged 12–14	Layered interventions through girl only clubs addressing multiple needs and risk behaviours of participants at personal, social, and economic levels	High participation among very young adolescents	*High* – Implemented in 2 health facilities in 2 rural southern districts, Zomba and Machinga	Increased knowledge of health issues Positive changes in gender norms and behaviour Increased peer support and social networks Income support to parents facilitated return to school of participants Increased self-efficacy to access HIV testing services

*PrEP – Pre-exposure prophylaxis for HIV; **ART – Antiretroviral therapy; ***AGYW – Adolescent girls and young women.

*Quality assessment* of the eligible studies was done using a critical appraisal tool for systematic reviews developed by Hawker et al,^42^ which is applicable to both qualitative and quantitative data, and includes a validated scoring system. Each component was given a score ranging from 1 to 4, using the criteria shown in Supplemental Table 2, for a possible maximum total of 32. Four authors completed the rankings, and one team member (FA) consolidated them. The Covidence platform was useful, not only in searching and identifying articles for screening for our systematic review but also in providing an option to tag articles that were ineligible but contained information relevant to our topic (see details below).

*Thematic analysis* was conducted by jointly selecting the main themes or categories of investigation in the selected studies through reviewing the Covidence reports on each article as a team and arriving at a consensus, and then searching each article to ensure that we had not missed any relevant information (see below). Eleven main themes (knowledge/understanding of SRHR, youth-friendly health services, adherence, addressing multiple needs, peer support, gender norms and relations, privacy, autonomy, stigma, hope for the future, decision-making power/agency) and several sub-themes were identified. These were divided among the four authors who searched all selected articles and inserted their findings by colour code into a joint Word document. These inputs were reviewed by the team and any additional information that had emerged from any of the searches was added. Thus we had a complete overview of each theme and how it was addressed in the studies from the perspective of each author. The four authors’ findings were then compared to each other to ensure consistency and completeness. These themes and sub-themes comprised our main findings which were then summarised for reporting in the Results.

*Intervention effectiveness* of participatory, health system-based interventions to improve ASRHR was assessed by WHO’s indicators of adolescent-friendly health services^43^ (see also Table 3): *equitable* (all youth are able to obtain the health services they need), *accessible* (youth are able to access the services provided), *acceptable* (health services meet the expectations of their clients), *appropriate* (health services that youth require are provided), and *effective* (services make a positive contribution to the health of adolescents). This was jointly assessed and discussed by the co-authors, and any discrepancies resolved together. For example, terms like “acceptable” and “appropriate” were sometimes confused and required further clarification and refinement, using the definitions above.

**Table 3.** Impact of desired changes on outcomes by WHO’s indicators of adolescent-friendly health services

Reference	Type of intervention	Desired change	Outcome				
			Acceptable	Equitable	Accessible	Appropriate	Effective
Nakalega et al, 2023	Peer support for HIVST and PrEP	Feasibility and acceptability of the peer approach	Yes	Yes	Yes	Yes	Yes*
		Adherence to HIVST	Yes	Yes	Yes	Yes	Yes*
		Adherence to oral PrEP	Yes	Yes	Yes	Yes	Yes*
Stangl et al, 2021	“Tikambisane”Curriculum delivered by support group	Increase in knowledge	Yes	NA	Yes	Yes	Yes
		Adherence to ART	Yes	NA	NA	NA	No*
		Stigma reduction	Yes	NA	NA	NA	No*
		Hope for future	Yes	NA	NA	NA	Yes
Mavodza et al, 2021	3 approaches for HIV testing assessed	(1) provider testing	Yes	NA	Yes	Yes	Yes*
		(2) self-testing on-site (no provider)	No	NA	No	No	No*
		(3) self-testing off-site (no provider)	No	NA	No	No	No*
Manda et al, 2021	Layered intervention (Girls only clubs within DREAMS)	Experience with clubs	Yes	NA	NA	Yes	Yes
		Change in knowledge of SRH	Yes	NA	NA	Yes	Yes
		Change in gender norms and behaviour	Yes	NA	NA	Yes	Yes
		Multiple needs met	Yes	NA	NA	Yes	Yes

Yes* – Indicator was measured quantitatively and positive changes occurred; Yes – Indicator was assessed qualitatively and positive changes occurred; No* – Indicator was measured quantitatively, and no impact was found; No – Indicator was assessed qualitatively, and no impact was found; NA – Not assessed quantitatively or qualitatively.

### Researcher reflexivity

The researchers were mindful of reflexivity throughout the preparation of this article, both individually and as a team. Now based in Ontario, Canada, we have diverse geographic and cultural roots, including from Africa, Central Asia, South Asia, and Canada. Team members were also at different stages in their career paths, from students to university professors. Four team members (CV, KA, AK) have extensive experience and current involvement in research, training, and humanitarian work globally, including in many African countries. The remaining co-authors were completing their undergraduate and graduate degrees at the University of Ottawa and McMaster University. We combined medical, social science, demographic, and library science expertise. We held weekly meetings online and in-person that were open to all team members. These provided an opportunity for regular reflection on our respective positionalities, such as how they shaped our research, and how these may have contributed to its richness and inclusiveness, as well as on areas of potential biases.

This article being a systematic review of existing literature and data already collected elsewhere, we did not feel that our positionality was likely to affect our analysis or findings, but we were aware of potential bias in our interpretations of them. Also, the article pertains to adolescent girls and young women in Africa who were not directly represented within our research team. For this reason, we benefited from the presence and contributions of our African co-author (KA) who is deeply sensitive to sexual and reproductive health and rights of African women, especially the often invisible and stigmatised diseases such as FGS.

As the final number of papers eligible for inclusion in this systematic review was small, we formed a sub-group (led by an early career researcher (FA)) to review and discuss all papers tagged as potentially interesting for context, and these provided important additional material relevant to the findings (see Results section). The final paper was developed through iterative meetings and circulation of working and final drafts.

## Results

### Articles selection

Our peer-reviewed search (illustrated in Figure 1) yielded 6225 articles from online databases which we imported to Covidence. Twenty-two duplicates were manually identified and removed, while 2597 duplicates were identified by Covidence and removed, leaving 3606 articles that were screened by title and abstract by two authors each. 3545 articles that did not meet our inclusion criteria were removed, including 24 that were posters or conference abstracts with no available full texts. Sixty-one full text articles were screened by two authors each. Only four articles met our eligibility criteria. Table 2 summarises the salient characteristics of the four included articles. Supplemental Table 1 provides all details, including title and full citation, reviewer, country where the study was conducted, objectives, methods (study design, start and end dates), sampling and data collection (type of sampling, description of data collectors, language, length of interviews, approach and content of interviews, data analysis methods, possible conflicts of interest for study authors), total number of study participants, their sex and age, inclusion and exclusion criteria for participating in the study, whether data presented on younger participants, description of intervention, type and degree of study subject participation, type and degree of health sector involvement, outcome and impact measures, intervention effectiveness, whether any focus on vulnerable groups, whether health workers’ perspectives were included and how, and ethical considerations.**Figure 2.**
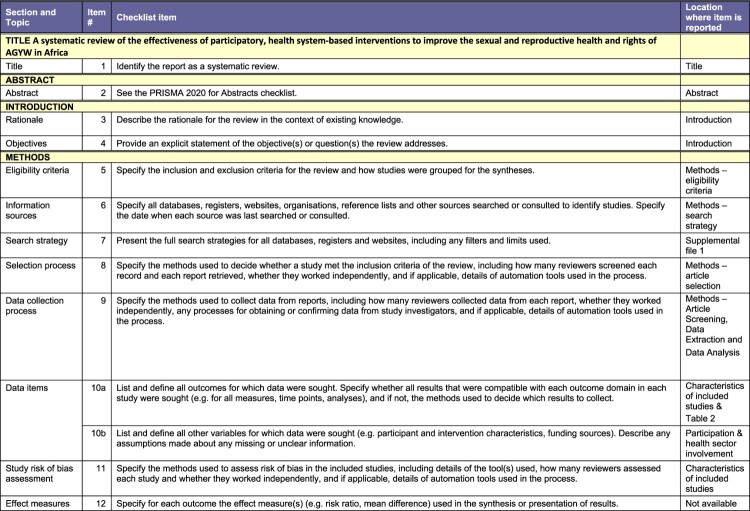
PRISMA checklist

**Figure 2b.**
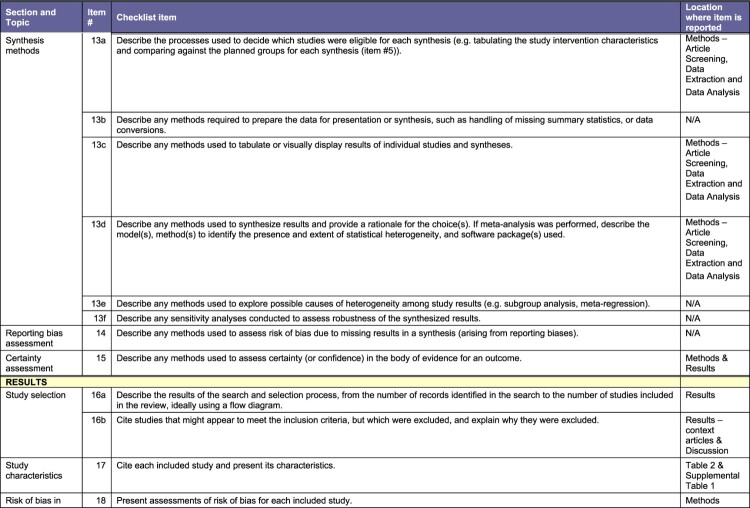
*Continued*.

**Figure 2c.**
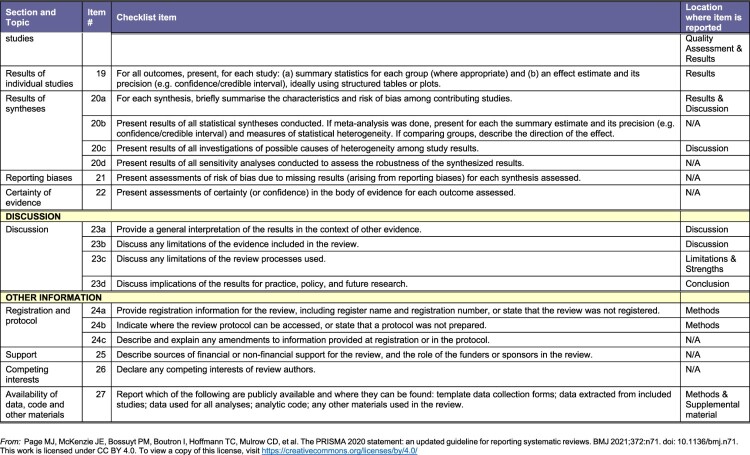
*Continued*.

Four kinds of interventions were described in the selected papers, a curriculum approach in Zambia,^44^ layered interventions in Malawi,^45^ peer support for HIV prevention in Uganda,^46^ and HIV testing models in Zimbabwe.^47^ Both the Zambia and Uganda studies involved peer support, whether to facilitate the learning sessions in the former^44^ or to provide information about HIV self-testing (HIVST) and PrEP. As noted above, quality assessments were made by four authors (see Supplemental Table 2) whose ratings were very similar – there were only four points difference between them. As a result, we do not prioritise our reporting of the results based on these ratings.

The articles tagged in Covidence as “refer for context” were ineligible for our systematic review for different reasons: they focused on interventions from sectors other than health (education, civil society), did not disaggregate findings by sex, or were not participatory in nature. For example, an article meeting most eligibility criteria, such as a participatory intervention for our target age group of AGYW, but delivered by an NGO (not the health sector), or an intervention delivered by the health sector but non-participatory in nature, was considered to merit consideration as part of a broader literature review in providing contextual information for our selected articles. A total of 276 articles were tagged as “refer for context” and reviewed by title and abstract by one of four authors (FA, PR, CV, LD), of which 75 had findings relevant to our research question. All relevant findings were extracted in Excel format (see Supplemental Table 3) and considered, as appropriate, in our Discussion section.

### Characteristics of included studies

The salient characteristics of the four selected articles are summarised in Table 2 (and detailed in Supplemental Table 1). They are briefly described below.

#### Study designs, populations, and outcome measures

The Zambia study evaluated a pilot support group curriculum, “Tikambisane” (“Let's Talk to Each Other”), for 21 HIV-positive AGYW,^44^ using participatory methods to increase knowledge about HIV, treatment with ART, and to help them cope with their positive HIV status. The Tikambisane curriculum was designed collaboratively through formative research with HIV-positive AGYW, educators on HIV stigma-reduction, health providers, HIV counsellors, program implementers, and research staff to identify the needs and concerns to be addressed in the program. Participants who were currently in HIV care were recruited from two Lusaka health facilities by the study coordinator with the assistance of clinic staff. The participatory methods included buzz groups, card storms, small group work, and role play, intended to increase knowledge and understanding of several HIV-related issues. The design included pre- and post-intervention surveys of clients, eight participant observations of the support group sessions, 17 in-depth interviews, and three other pre- and post-intervention tools to measure changes over time: a Stigma Scale for Chronic Illness, a Hope for the Future Scale, and the CASE (Center for Adherence Support Evaluation) Adherence Index for assessing antiretroviral therapy (ART) adherence. The in-depth interviews explored sources of support, challenges experienced living with HIV, how HIV and ART affected the AGYW’s bodies, life aspirations, and hope for the future. Analysis included descriptive statistics at baseline for the 21 AGYW who participated in the intervention, while analyses comparing baseline and endline outcomes were restricted to participants who had data at both time points (*N* = 14). This study only marginally met our inclusion criteria because the intervention was conducted outside nearby clinics by non-clinic staff and peers, but we decided to include it because it was co-designed with Ministry of Health representatives and health workers who assisted with some of the support group sessions.

The Malawi study reported on a “girls only clubs” intervention for a population of 23 very young adolescents (VYA) aged 10–14 in two rural districts in southern Malawi, using layered interventions (addressing multiple needs)^45^ The interventions used a central principle of addressing health and socio-economic challenges at individual, community, structural, and socio-physical levels through girls only clubs.^45^ They included primary interventions such as social asset building, HIV testing services, and condom information, and secondary interventions such as village savings loans for caregivers and back-to-school support, among others. The club facilitators used the DREAMS (Determined, Resilient, Empowered, AIDS-free, Mentored and Safe) tool kit,^45^ a curriculum to train the AGYW on skills associated with SRHR and other life challenges. Located in community-based safe spaces, the clubs use participatory learning approaches, such as discussions, games, and role-plays, to build social assets and life skills. The study used purposive sampling to identify girls who had participated in the clubs for about 12 months, and a narrative inquiry approach, involving unique stories from the participants, to assess the impact of the intervention, a method the researchers considered appropriate for VYA. The outcome measures were qualitative, based on the accounts of the VYA about their experiences as participants in the clubs, as well as changes in the SRHR knowledge and other social and economic empowerment indicators.

The Uganda paper described a pilot study to investigate the feasibility and acceptability of peer-delivered HIV self-testing and PrEP as HIV prevention strategies among a random sample of 30 AGYW aged 18–24 from a pool of AGYW at one health centre in Kampala.^46^ Study participants were offered daily oral PrEP and attended regular clinic visits, between which they were visited monthly by trained peers who delivered HIVST kits and PrEP, and provided adherence counselling with drug-level feedback. Lay counselling and bi-weekly telephone support were also provided by peers. No control group was included for comparison. Although some participants were older than our eligible age spectrum, we opted to include the article because nearly half (47%) were aged 18–19 (within our eligibility range) and several findings were reported by age, allowing us to focus on the results for that sub group.

Participants who had been on PrEP for at least one month and had suboptimal adherence were selected from 60 AGYW receiving PrEP at the health centre. The study nurse performed HIV testing and took urine samples at baseline, 3-month, and 6-month clinic visits. Three peers (aged 18–24) who had strong adherence to PrEP were purposively selected from the health facility. The study included both quantitative outcome measures to evaluate participants’ experiences with intervention delivery – comparing actual versus planned product delivery and product use – and qualitative information through two focus groups with AGYW and five in-depth interviews with peers and health workers. The freedom and independence of the study participants to conduct self-testing and PrEP were considered as participatory aspects of this study, as well as their informal feedback to peers and health workers throughout the project.

The Zimbabwe paper investigated preferences for HIV testing (provider testing, in-clinic on-site self-testing in a private booth alone, and off-site self-testing) in a sample of 700 girls aged 16–24, of whom 472 (50%) were aged 16 to 19. This study included males as well as females, but for our analysis, we concentrated on the results pertaining to females aged 16–19. It collected both quantitative and qualitative data. The quantitative results, reported for the whole sample, were complemented by qualitative information from a convenience sample of 31 participants, 26 of whom were females 16–19 years old. Eight in-depth interviews were held with health providers and clients and four focus group discussions with clients (two female, one mixed and one male). The quantitative outcome measure was the choice of HIV testing method, and the qualitative outcome measures were reasons for their choice (confidence in type of testing selected, importance of a youth-friendly environment, and barriers to testing alone). The participatory aspect of this study was the most limited of the four papers, but because the participants had the autonomy to choose the kind of testing they preferred and could freely self-test on their own (as in the Uganda study), we considered it to meet our definition of the term.

#### Ethical considerations

All studies were conducted with formal approval of the investigating institutions. The purpose of the respective studies was explained to participants in all settings and confidentiality and anonymity were strictly observed. Written informed consent was obtained in all studies. In Zambia, in conjunction with the health services, participants were excluded if they were considered mentally unavailable or at high risk of abuse from participation in the study, or whose parent/guardian was unaware of the young woman’s HIV positive status. For younger participants (aged 12–14 in Malawi and those under 16 in Zimbabwe), verbal consent was taken, as well as the written consent of parents or guardians. In Uganda, informed consent for illiterate participants was taken in the presence of an impartial witness.

#### Intervention effectiveness

As noted above, all four studies evaluated adherence to the specific interventions selected. Table 3 assesses each type of intervention, desired changes, and the outcomes described in the four articles using the WHO benchmarks mentioned above. Their strengths and weaknesses are discussed in the following sections.

### Curricula using participatory methods in Zambia

Qualitative data on the Tikambisane support group intervention in Zambia^44^ indicated that participants felt they gained knowledge from the curricula, and that it boosted their confidence, their acceptance of treatment, and strengthened their hope for the future. However, none of the quantitative indicators (adherence to ART, stigma reduction, and hope for the future) showed any statistically significant change as a result of the program (Table 3). It is therefore difficult to reach a definite conclusion about the effectiveness of the intervention, given that indications of positive impact from the qualitative data were not substantiated by the quantitative findings. The intervention was highly participatory, but the involvement of the health system was the weakest of all the studies.

### Layered interventions in Malawi

The study’s impact, as measured by VYAs’ perceptions, experiences, and their reported influence on practical learnings about health issues as a result of club participation, was positive (Table 3). The clubs served as a safe space for AGYW to learn about STIs and HIV prevention, contraception, menstrual hygiene, improved access to health services, educational support, social skills, asset building, and economic strengthening through promoting village savings loans for parents and providing school learning materials and uniforms. Several participants who had dropped out of school said they decided to return after listening to motivational talks by other participants. Also, they reported that they were empowered to confront inequalities in their lives, such as raising issues with family members like overworking children, keeping girls home from school to care for younger siblings, unequal household burdens for girls compared to boys, or forcing girls to marry against their will. The authors considered the layering of interventions to be key benefits with more positive impact than single interventions alone. Although the data presented were qualitative, the methodology seemed appropriate for the study’s very young population, and the intervention seems to have been effective in achieving its results. Additionally, it was highly participatory and health system based.

### Peer support in Uganda

The effectiveness of the approach was assessed by three dimensions: feasibility and acceptability (comparing actual versus planned delivery of the intervention and product use); adherence to HIV self-testing (sharing results with peers during their visits); and adherence to oral PrEP (measured by urine tests). The study found that peer support seemed to enhance adherence and motivated participants to remain in the study throughout. The high level of compliance was attributed partly to a large number of participants who were sex workers (60%), all of whom were adherent to PrEP. Privacy and confidentiality were also mentioned by participants as positive aspects of peer support, thus avoiding potential stigmatisation or risk of disclosure at crowded public clinics. Participants also expressed appreciation for saving travel time and costs for clinic visits, the drug-level feedback, adherence counselling, the openness of peers to discuss clients’ concerns, and the educational information and encouragement provided. Peer support was also considered more equitable than that of health clinics. Health providers also indicated that peers, who were familiar with the sexual challenges facing AGYW, were valued intermediaries between providers and clients throughout the program. This study provides strong evidence of the intervention’s effectiveness, using the WHO criteria for AFHS (Table 3). Moreover, it was conducted with full participation of the study subjects and a local health facility.

### Comparing HIV testing options in Zimbabwe

In the Zimbabwe paper^47^ comparing different HIV testing options, both quantitative and qualitative indicators showed a strong preference for provider-delivered HIV testing (Table 3). Participants cited confidence in the providers' expertise, the pre- and post-test counselling received, and the privacy afforded by the clinic. They also praised the provider’s reassuring support throughout the testing experience, the advice received on care and treatment, and how to share a potential positive result with family members. Conversely, participants were doubtful about their ability to self-test, interpret the results correctly, or cope with a positive result. While participants emphasised the importance of discretion in HIV testing, they stressed that this did not necessarily entail undertaking the test on their own. The authors concluded that reaching AGYW for HIV prevention can be augmented through services that are reinforced by an ethos of acceptance and support. In terms of effectiveness, the provider-initiated intervention was considered effective in meeting the needs of the clients because of their trust in the providers’ expertise, among other reasons. The participation of the study population was limited in the case of the larger sample, but high for the smaller group who provided qualitative feedback. There was full involvement of the health sector.

## Discussion

This systematic review aimed to assess the effectiveness of participatory health system interventions in improving the sexual and reproductive health and rights of adolescent girls in Africa. Despite the large body of research reviewed, only four articles addressed our research question on participatory health sector-led interventions. Most other papers focused on interventions developed by NGOs and other sectors, or on adolescents as recipients, rather than as full participants in the research process.

Our four included studies all aligned with Freire’s principles and approach to social change: promoting power-sharing, critical consciousness, dialogue, and praxis. For example, in Uganda participants appreciated the non-judgmental manner in which peers shared information, the equitable dialogue engendered between them, and their own heightened empowerment to protect themselves from HIV. Praxis, another Freire approach, was demonstrated in the studies to different degrees, such as sustained adherence to PrEP in Uganda^46^ and increased motivation to undergo HIV testing in Zimbabwe.^47^ In Malawi, girls club participants successfully advocated for increased sharing of domestic roles with boys in their households as a result of the agency fostered by the intervention.^45^ In Zambia, the participatory training in various aspects of coping with HIV appeared to promote self-efficacy and acceptance, at least based on the qualitative findings.^44^

Attention to adolescent rights was inherent in the research approaches in all included studies (full explanations of the research, written informed consent). However, the only study that clearly adopted a rights perspective was the Malawi paper, which promoted gender equality, agency, the right for girls to attend school, and confronting child abuse, among others.

A salient finding of this review is that, contrary to widespread evidence that health providers are often barriers to health seeking among AGYW in Africa,^44,48–51^ health staff were widely considered to be trusted professionals whose knowledge and support were valued over that of others, provided that they were non-judgmental and treated adolescents’ concerns confidentially and within a private setting. Privacy has been widely evidenced elsewhere as critical to youth-friendly health services.^24,35,52–55^ Interestingly, while HIV self-testing was originally developed as an attractive option for hard-to-reach populations, participants in the Zimbabwe study considered it to be a barrier to testing because they lacked confidence in their ability to perform the test properly. Similarly, in the Uganda study,^46^ the back up of health professionals was an important element in the intervention’s roll out, although participants felt more comfortable discussing their personal problems and concerns with knowledgeable peer counsellors. Thus adequately trained and sensitive health workers can be key to meeting the unique needs of young people, in addition to other trusted stakeholders, such as peers.

Another important finding of this review was the value that AGYW attached to learning about SRHR and life skills, especially in the girls only clubs where knowledge sharing was a main objective of the intervention. Information from health workers was particularly trusted, but additional guidance from peers appropriately trained in HIV prevention was also valued. School SRHR programs were less appreciated than the more comprehensive information gained from health staff and qualified peers. Similar findings were reported in Northern Uganda^56^ and Zimbabwe^57^ where the provision of menstrual health information and products by health services was positively received by young women. Studies of adolescent needs in Zambia found that AGYW wanted more information from health staff on safe delivery and early childcare,^58^ and on cervical cancer screening.^59^ Other studies have also found that health services that incorporate agency building into their programs can enable AGYW to withstand negative influences in their environments, including harmful norms and gender barriers, and achieve better health outcomes.^35,53,60^ Again, health workers are uniquely positioned to provide this information when they are trained and empowered to do so.

Similarly, layered interventions, addressing multiple needs at different levels of society, were found to be useful in promoting the wellbeing of AGYW beyond SRH alone. In the reviewed literature, including the contextual articles, many intersectional factors affecting ASRHR were identified, including gender inequality, financial insecurity, and lack of education, autonomy, and decision-making power. Gender power relations, such as “sugar daddy” pressures on younger females, the fear of violence if refusing to comply, and the tension between traditional ideals of female chastity and modern ideals of sexual freedom, were frequently cited in the broader literature as reasons for succumbing to unwanted sex, greater exposure to HIV and STIs, and problems accessing VCT,^35,46^ particularly in resource-constrained environments.^61^ Contextual factors were also found to influence compliance with HIV viral suppression advice among youth seeking YFHS in Nigeria^53^ and access to menstrual health products in Kenya.^62^ Thus it seems that adolescent girls are likely to prefer more comprehensive programs encompassing responses to the many aspects of their experiences and challenges as young African youth, in addition to more biomedical SRH information.

Peer support has been used widely as an intervention to engage AGYW, especially in HIV programs.^63^ Positive evidence for its added value was seen in all selected papers. In these studies, peer involvement was useful in promoting knowledge and attitude change, especially when peers were personally affected by the issue being addressed, such as being HIV positive in the Zambia study,^44^ or when peers were seen as well informed and trustworthy, as in the promotion of HIV self-testing and PrEP in Uganda.^46^ Similarly, social connections have been found to enhance health outcomes and overall wellbeing, and in reducing isolation and stigma among vulnerable groups, including peer navigators, in South African primary health clinics,^64^ Project YES! in Zambia,^65^ and among refugee youth in Uganda.^31^ Nonetheless, the only one of our included articles to quantifiably measure stigma reduction (Zambia),^44^ found no significant behavioural impact of the peer-supported curriculum intervention. Further, a recent overview of systematic reviews of peer-based interventions found that while they helped improve SRH knowledge and attitudes, their effectiveness in promoting actual behaviour change was unclear.^63^ Another systematic review of sex education interventions to improve unintended adolescent pregnancy, intimate partner violence, and FGM/C among adolescents globally found that peer-led counselling significantly improved knowledge but did not significantly impact contraceptive use,^66^ and a longitudinal study of the influence of peer friendships on preventing adolescent pregnancy among girls in a poor Nairobi settlement found no independent influence on early pregnancy, while friendships with out-of-school males increased pregnancy risk significantly.^67^

The paucity of attention to the rights component of SRHR in the adolescent health literature is a concern, given that the protection of women, including AGYW, from any interference in their fundamental freedoms and human dignity is enshrined in the Universal Declaration of Human Rights and subsequent international conventions. Health services based on human rights principles ensure fully informed decision-making, respect for dignity, autonomy, privacy and confidentiality, and sensitivity to the needs and perspectives of individuals.^68,69^ The need for a stronger human rights agenda in ASRH interventions has also been noted elsewhere,^63,70,71^ including in South Africa^51,68^ and Uganda.^72^ Moreover, detailed guidelines are available to inform rights-based gender-sensitive approaches.^73^ This is an area where schools and NGOs can play a complementary role to health services, such as in the girls only clubs^45^ and other community-based programs.^51,74,75^

Although HIV is a major concern for African youth, the attention it receives in the AGYW-focused literature seems disproportionate to their other needs, as noted elsewhere.^63^ These needs include information and guidance on menstrual health, early pregnancy and childbearing, domestic and inter-partner violence, FGM, and FGS. In many African settings, health workers lack comprehensive knowledge about the range of diseases and conditions that affect ASRHR, such as neglected infectious diseases. For example, FGS impacts up to an estimated 56 million girls globally,^12^ mainly in Africa, yet most health workers are not trained to recognise and diagnose the disease.^76,77^ Moreover, effective treatment programs, such as mass drug administration of praziquantel, largely fail to reach adolescents because they are provided mainly to younger, primary school-aged children.^78^ For adolescent girls especially, symptoms are often confused with those of STIs, resulting in fear, stigma, and hesitancy to seek treatment.^70^ Integrating FGS into existing ASRHR policies and services, such as providing praziquantel therapy alongside HIV treatment in areas where schistosomiasis is prevalent,^79^ could significantly alleviate suffering and offer more comprehensive SRH care for adolescent girls.

## Limitations and strengths

The main limitations of this systematic review are the small number of research papers eligible for inclusion, the small sample sizes in all but one study (Zimbabwe), and the fact that most of the findings reported are qualitative. Although quantitative measures of effectiveness were used in three studies, all articles relied heavily on qualitative data to interpret and validate their findings. More studies using rigorous research methods and robust sample sizes are therefore needed to further investigate and validate the findings in this review.

A further limitation is that the included studies are not easily comparable because of their different aims, methodologies, and study populations. For example, the focus of the Malawi study on empowerment of VYA, including in SRHR, was very different from that of the Zambia paper on a curriculum for older, HIV-positive adolescents. These populations were again different from the Uganda participants, a large number of whom were sex workers. Also, where the study population included participants outside our eligibility criteria (older AGYW or males) we tried to focus on only those results pertaining to AGYW aged 10–19, but it is likely that some findings were also attributable to the broader female study population as well. Additionally, the nature of the interventions was different in several respects, complicating comparisons of their relative effectiveness. The participatory approaches were also distinct and more limited in some contexts than others. Thus conclusions about the relative strengths and weaknesses of the different approaches used in the interventions remain tentative.

Another limitation is that, while a key criterion for inclusion in our review was the involvement of the health system, its role and visibility varied considerably in the four selected articles, from minimal presence in Zambia to a supportive role in Uganda and Malawi, to significant involvement in Zimbabwe. Moreover, we were unable to find much information in the included studies on the constraints and opportunities for health services in providing YFHS to their clients, which remains a gap in our knowledge. Given its centrality to the interests of this review, the role and added value of the health sector could have been more fully articulated in the studies.

The primary strength of this review is that it identified an important research gap that the selected studies are helping to fill. Despite our exhaustive literature search for interventions to improve the SRHR of AGYW in Sub-Saharan Africa, very few have been documented within the context of African health systems, and even fewer with a participatory, rights-based approach. The included studies demonstrate the importance attached by AGYW to being included in the interventions as active contributors and not merely as passive recipients. Finally, the recognition of the rights component in SRHR is a further strength of the selected studies in this review.

## Conclusion and recommendations

To conclude, the promising interventions in this paper support the view that participatory health system-based interventions can improve the SRHR of adolescent girls and young women in Sub-Saharan Africa, and that investing in Sub-Saharan African health systems by global health and development efforts would seem preferable to supporting parallel initiatives delivered through other sectors. These investments should focus on the training and sensitisation of health workers in YFHS, supported by essential resources, supplies, and basic infrastructural improvements to facilities to accommodate privacy considerations. Seemingly small investments in health worker capacitation and sensitisation to the needs of AGYW, including comprehensive SRHR information, could have a positive and sustainable impact on their ability to confront and mitigate the multiple challenges they face in their lives.

For AGYW in the selected studies, the support of knowledgeable peers appeared to increase confidence in, and uptake of, the interventions, although their added value was difficult to quantify. Health staff also seemed to appreciate their contributions. These findings suggest that integrating peer support into quality health services, where appropriate, can contribute to emotional wellbeing and health outcomes for AGYW. Finally, our findings validate the importance of subject participation in the interventions and research designed to help them, both as rights holders and as beneficiaries. While the type of intervention and degree of participation varied in the four studies, we can identify several important aspects of the participatory approach that were common to most of them. First, from the perspective of the AGYW themselves, participation was motivated by anticipated personal benefits, such as learning about protection from HIV, sexual health, or coping with difficult family circumstances. Second, they were motivated by interventions that promoted equitable relationships, such as the involvement of friends or peers, or other participants of similar age and gender, whom they felt understood their situations and could help them express issues of concern, such as their fear of HIV testing or how to share confidences with health workers. Third, engagement of the participants in their care and wellbeing was sustained by gaining information that they valued and could use in their lives, as well as skills learned such as how to negotiate gender challenges within their families. Fourth, implementing an intervention in an atmosphere of respect and trust helped sustain participation, including when peers and/or health providers were seen as imparting valuable knowledge and able to maintain confidentiality. Flexibility on the part of the implementers was also valued by participants, allowing them to have a voice in the activities they preferred or the timing of their participation. Finally, locating the intervention in a familiar context, such as within the participants’ communities, seemed to help motivate and sustain their participation in the intervention.

Several recommendations flow from these conclusions. First, as also noted by others,^2,56^ health services for AGYW should foster a supportive and engaging environment that meets the unique needs of young people, tackles logistical barriers, and incorporates the perspectives and developmental needs of adolescents. In this respect, YFHS guidelines or protocols can be an important resource, although they are not consistently available in African health facilities.^80,81^

Second, interventions that provide comprehensive integrated SRHR education, not limited to HIV and STI issues only, have considerable potential for reaching this demographic.^59,82,83^ They can attract VYA as an entry point for instilling important life skills and self-confidence, including in education, gender equality, SRHR, and economic agency. Such interventions can benefit from the collaboration of different sectors in conjunction with adolescents themselves, in their design and implementation. This is an area where schools and NGOs can play a complementary role in health programs, such as in the girls clubs^45^ and other community-based programmes.^51,74,75^

Finally, identifying the discrepancies between international commitments to adolescent and child rights and existing social norms and practices at different levels of society, through reflection, mobilisation, and collective multisectoral action, can help develop positive new norms in support of ASRHR.^84^ Taking stock of shortcomings and identifying positive ways to mitigate them, including investing in adolescent-friendly health sector interventions, is increasingly important and timely as countries are asked to report on their progress toward the targets of The Global Strategy for Women’s, Children’s and Adolescents’ Health (2016–2030).

## Author contributions

*Conceptualisation: CV, AK. Study design: CV, PF, LD, CC, AK. Formal analysis: CV, FA, LD, PR. Funding acquisition: AK. Methodology: PF, CV. Investigation: CV, FA, LD, PR, CC, RS. Writing – original draft: CV, FA, LD, PR, KA. Review & editing: AK*.

## Supplementary Material

Supplemental Table 1. Detailed data extraction information - included studies.

Supplemental File. Search strategies and included African countries.

Supplemental Table 3. List of context articles for full text review.

Supplemental Table 2. Quality assessments of four included studies.
