# Supplementary material for: A systematic review of the effectiveness of participatory, health system-based interventions to improve the sexual and reproductive health and rights of adolescent girls and young women in Sub-Saharan Africa
Source: Sex Reprod Health Matters. 2026 Mar 18;33(1):2643037. doi: 10.1080/26410397.2026.2643037 (PMC13103997; doi:10.1080/26410397.2026.2643037)
Supplement: Supplemental Table 1. Detailed data extraction information - included studies. [file ZRHM_A_2643037_SM3933.docx]

| **Supplemental Table 1: Detailed Data Extraction Information - Included Studies** | | | | |
| --- | --- | --- | --- | --- |
| **Study characteristic** | Stangl AL, Mwale M, Sebany M, Mackworth-Young CR, Chiiya C, Chonta M, Clay S, Sievwright K, Bond V. Feasibility, Acceptability and Preliminary Efficacy of Tikambisane ('Let's Talk to Each Other'): A Pilot Support Group Intervention for Adolescent Girls Living with HIV in Zambia. J Int Assoc Provid AIDS Care. 2021 Jan-Dec;20:23259582211024772. doi: 10.1177/23259582211024772. PMID: 34212766; PMCID: PMC8255553 | Manda, W.C., Pilgrim, N., Kamndaya, M. et al. Girl-only clubs influence on SRH knowledge, HIV risk reduction, and negative SRH outcomes among very young adolescent girls in rural Malawi. BMC Public Health 21, 806 (2021). https://doi.org/10.1186/s12889-021-10874-x | Nakalega, R., Mukiza, N., Menge, R. *et al.* Feasibility and acceptability of peer-delivered HIV self-testing and PrEP for young women in Kampala, Uganda. *BMC Public Health* **23**, 1163 (2023). https://doi.org/10.1186/s12889-023-16081-0 | Mavodza CV, Mackworth-Young CRS, Bandason T, Dauya E, Chikwari CD, Tembo M, Apollo T, Ncube G, Kranzer K, Ferrand RA, Bernays S. When healthcare providers are supportive, 'I'd rather not test alone': Exploring uptake and acceptability of HIV self-testing for youth in Zimbabwe - A mixed method study. J Int AIDS Soc. 2021 Sep;24(9):e25815. doi: 10.1002/jia2.25815. PMID: 34569710; PMCID: PMC8474521 |
| **Objectives** | To assess the feasibility, acceptability and preliminary efficacy of a 6-session support group intervention designed to facilitate healthy transitions to adulthood among AGYW aged 15-19 living with HIV in Lusaka, Zambia. | To assess: 1. Perceptions & experiences of very young adolescent (VYA) girls on Girls-only club participation in 2 rural southern districts of Malawi. 2. Impact of club participation on VYA’s sexual and reproductive health (SRH) knowledge to reduce risk for HIV and negative sexual health outcomes | To assess the feasibility  and acceptability of peer delivered HIVST & PrEP to young women in Uganda. | To investigate the uptake and acceptability of different HIVST testing options in Harare, Zimbabwe, embedded in the CHIEDZA cluster randomized trial. |
| **Start & End Dates** | 2015 to 2018 | 07/2017 to 05/2019 | 03/2021 to 09/2021 | 04/2019 to 0/6 2019 |
| **Sampling & Data Collectors** | Purposive sampling; Pre-post, Mixed methods design. A trained team, consisting of an adult HIV counselor & participatory educator & 2 peers living with HIV facilitated each session. 12 peers were involved in developing the support group curriculum, & participated in a 2-day training workshop on listening & facilitation skills & support group curriculum content | Purposive sampling. Interviews were conducted by a female researcher with experience of working with adolescents. | Systematic random sampling from a pool of individuals at one health centre. Sample of 30 AGYW aged 18-24 years, who were on  PrEP for at least one month & had suboptimal adherence was selected from the 60 AGYW receiving PrEP at study site. The study nurse performed HIV testing. Structured questionnaire for quantitative part & qualitative interviews done by trained social scientist | Convenience sample of 35 youth from all youth (n=1414) who had taken an HIV test at the site within the previous 11 weeks, with variations in age and sex. 31 participated and 26/31 were female, most of whom were 16-19 years Trained.qualitative researcher conducted focus group discussions (FGDs) & IDIs in Shona language, using a method-specific topic guide. |
| **Language** | Bemba, Nyanja, or English | Chichewa | English & Luganda. Participants were interviewed in their preferred language. | Shona |
| **Interview Length** | In-depth interviews (IDIs) took 60 minutes & surveys took 45 minutes | 30-4- minutes | Each interview 25-50 min. FGDs 40-60 min | IDI – 60 min  Surveys 45 min |
| **Approach & Content of Interviews** | Surveys: 14 item Stigma scale for Chronic illness used to measure stigma, 12 item Hope Scale used to measure hope for future, ART adherence measured using CASE adherence Index  2. IDI: Narratives during pre & post intervention explaining existing sources of support, challenges experienced due to living with HIV, and the impact of both HIV & ART on the AGYWs body, life aspirations & hope for the future | Narrative inquiry approach collecting unique stories, interpreting their daily experiences & how they make use of wider social/cultural resources to make sense of their lives | Qualitative research, as part of multi-method study  Systematic random sampling from a pool of individuals at one health centre. Sample of 30 AGYW aged 18-24 years, who were on  PrEP for at least one month and had suboptimal adherence, was selected from the 60 AGYW receiving PrEP at study site | Quantitative pproach used to track client service usage across multiple visits, while qualitative data used to explore perceptions/experiences of healthcare providers & clients of the 3 HIV testing methods.  Qualitative approach - 4 client FGD - 2 female only, one male only, & one mixed. 2 paired female same-sex IDIs & 6 individual IDIs |
| **Data Analysis Methods** | Transcripts & notes coded in Nvivo 11.0 & analyzed by content analysis. Deductive coding & analysis used to assess feasibility, acceptability, & initial efficacy of intervention. Survey data analyzed in Stata14. Descriptive baseline statistics reported for the 21 intervention participants & baseline & endline comparisons reported for the 14 who completed both pre- & post-intervention | IDIs transcribed & translated (Chichewa to English). NVIVO 11 software using thematic analysis based on study objectives. 2. Quality assessed was conducted by checking the transcripts against the audios. An independent researcher tested the codebook to assess if there was inter-coder agreement | For feasibility, acceptability and adherence, quantitative measures used. For qualitative data analysis Atlas.ti version 8.3 was used. Data coded by 2 coders. The Consolidated Criteria for Reporting Qualitative Studies checklist was used to report study findings. | Quantitative measures used for feasibility, acceptability & adherence. Qualitative data analysis used Atlas.ti version 8.3. Data coded by 2 coders. The Consolidated Criteria for Reporting Qualitative Studies checklist was used to report findings. |
| **Participants’ Number, Sex, & Age** | 21 females aged 15-19 HIV in Lusaka who were currently in HIV care | 23 females aged 12-14 | 30 females aged 18-24 years who were on  PrEP for at least one month with suboptimal adherence | Quantitative HIV testing 951 individuals of whom 700 were female. 472 individuals were aged 16-19.  31 were included in qualitative portion of study, of whom 26 were female & 27 were 16-19 years old |
| **Inclusion Criteria** | Eligible participants included AGYW aged 15-19 years living with HIV in Lusaka who were currently in HIV care | 1. Age 12-14.  2 .Availability & willingness to participate, ability to communicate experiences & opinions in an articulate, expressive, & reflective manner.  3. Parent / guardian permission received & consent of participant obtained | 1. Females aged 18-24 years, who were on PrEP for at least on month, and had suboptimal adherence.  2. Peers: females aged 18-24 were purposively selected based on having no interruptions in their PrEP refills for at least one year, lived within the catchment area of the study site, & were literate | 1. Youth aged 16-24 years resident within cluster boundaries 2. Clients who did not know their status and/or had not been tested in the past 6 months were eligible for HIV testing |
| **Exclusion Criteria** | Those who were mentally unable, or at high risk abuse as a result of participation, or had a parent/guardian who was unaware of their HIV positive status, or had ever participated in a formal support group for youth living with HIV | Girls younger than 12 or who didn't meet other inclusion criteria | Younger adolescent girls & AGYW with optimal performance | 1.Those who took a kit for off-site testing could not be included as contact details were only for clinical follow-up & not for research purposes. 2. Those who hadn't taken an HIV test or refused one were excluded |
| **Type of Intervention** | Tikambisane (“Let's talk to each other”) curriculum using participatory methods to address HIV disclosure and stigma, ART, relationships, grief loss, & planning for the future | DREAMS - Girl-only clubs "Layered Interventions", including Primary interventions - (1) Social asset building, (2) HIV testing, (3) Condom information, (4) Screen for case management. Secondary interventions - (1) Combined socio-economic support approaches (Village savings loans) for caregivers, (2) Food security/ nutrition, (3) Post violence care (4) Access to contraceptive information/ services (5) Back to school support | Provision of PrEP for HIV prevention and HIV self test kits (HIVST). Peers provided PrEP adherence support and lay counselling (i.e., active listening, advice about side effects) during monthly visits in addition to bi-weekly telephone support. HIV testing was performed on urine samples taken by the study nurse at baseline, 3-month, and 6 month clinic visits  | Community-based intervention to improve HIV outcomes for youth. Three HIV testing options offered: (1) provider testing by trained providers (2) HIVST on-site in a private booth without provider present; & (3) provision of a test kit to test off-site. For all options, an oral mucosal test (OMT) was used and clients counselled that a reactive test would require confirmation by a blood-based rapid antibody test as per national guidelines |
| **Outcome/**  **Impact Measures** | Quantitative -1. Stigma: 14-item Stigma Scale for Chronic Illness  2. Hope for the future: measured using the 12-item Hope Scale 3. ART adherence: measured by the CASE Adherence Index.  Qualitative: Explored sources of support, challenges living with HIV, & physical & aspirational impact of both HIV & ART | 1. Experiences participating in the Girls-only Clubs  2. Change in the SRH knowledge to reduce risk of HIV and other negative sexual health outcomes | Acceptability of peer delivered PrEP & HIVST was 97% (29/30), and 93% (28/30) at the 3 and 6 month time points, respectively. All the participants performed their HIVST with the peer during the monthly visits | 1. Quantitative - Uptake of testing - 67.2% (n=951). Provider testing accounted for 94.4% of tests done. 74% were females.  2. Qualitative - Strong preference for provider testing as perceived as highest quality option. No gendered patterns in preferences & experiences  Confidence in youth-friendly environment  Face to face counselling - positive, felt supported in sharing a positive result with parents  Lack of confidence in own expertise in using oral test-kit  -lack of privacy at home  -fear of getting results alone  -on-site private testing took longer wait |
| **Intervention Effectiveness** | 1. Positive feasibility outcomes with full enrollment of 6-week baseline assessment period & good intervention participation noted. Positive acceptability outcomes with participants expressing their appreciation for co-facilitation including both an adult counsellor & peers living with HIV.  2. Mean scores on hope for the future scale increased slightly following the intervention, from a mean score of 3.0 to 3.2 (p = 0.261).  3. No significant increase seen in ART adherence following intervention.  4. Participants indicated that the session on ARVs enhanced their understanding of benefits of adherence, increasing their self-efficacy & acceptance.  5. Intervention appears to have met the expectations expressed by the participants | 1. Increased knowledge about health  2. importance of providing comprehensive & accurate sexual health information 3. Positive changes in gender norms 4. Positive role of parental support to reinforce norms 5. Facilitated peer support & social networks 6. Income support to parents enabled out-of-school girls to return to school 7. Enhanced agency to access health services, including HIV testing 8. Added value of "layered interventions" - addressing multiple needs of young people has more impact on risk behavior reduction than any single intervention alone | All participants accepted peer-delivered services at baseline & most still receiving them at study end. AGYW said they found peers friendly & approachable, services convenient & helped reduce transport costs, among other reasons. Peer relationships perceived as equitable compared to health worker relationships, helping motivate & improve PrEP adherence. Adherence counselling with drug-level feedback highly acceptable | 1.Testinig was undertaken by 2/3 of those eligible. 2. Youth overwhelmingly preferred provider testing because it was conducted at a trusted youth-friendly service within the community by expert staff, whom they anticipated would provide effective in-person support throughout the process. 3. Considerable hesitancy about HIVST. Shows that, contrary to narrative that youth primarily desire autonomy and privacy in health service engagement, where provider testing is non-judgemental & youth-friendly, this is the preferred option.  4. Pathway to improving uptake of HIV testing among this demographic may be through increasing investment in the provision of services which are underpinned by an ethos of acceptance & support |
| **Ethical considerations** | Written, informed consent gained from a parent/guardian for participants under 18, & directly from those 18+ prior to enrolment. Participants given a referral list of organizations offering physical, social, or psychological support. Ethical clearance from the International Center for Research on Women, Washington, D.C., London School of Hygiene & Tropical Medicine, University of Zambia Humanities & Social Science Research Ethics Committee, & the National Health Research Authority, Zambia. Study findings & curriculum disseminated to stakeholders & nationally | Ethical approval from College of Medicine Research Ethics Committee (COMREC) in Malawi, the University of the Witwatersrand Human Research Committee (HREC) in South Africa & from Population Council, USA. Agreements with several other international donors were signed. Before interviewing the VYA girls permission was obtained from a parent or guardian using an appropriate informed consent document | The study was approved by Makerere University School of Medicine Research Ethics Committee (2020-133) and the Uganda National Council for Science and Technology. Administrative clearance was obtained from Kampala Capital City Authority | Ethical approval was granted by the Medical Research Council of Zimbabwe, the Biomedical Research and Training Institute Institutional Review Board & the London School of School of Hygiene and Tropical Medicine Ethics Committees. Written informed consent was obtained from all interviewees. A waiver for the requirement of guardian consent was granted for 16-and 17-year-olds |
| **Conflict of Interest** | None stated | None stated | None stated | None stated |
