# Supplementary material for: A systematic review of the effectiveness of participatory, health system-based interventions to improve the sexual and reproductive health and rights of adolescent girls and young women in Sub-Saharan Africa
Source: Sex Reprod Health Matters. 2026 Mar 18;33(1):2643037. doi: 10.1080/26410397.2026.2643037 (PMC13103997; doi:10.1080/26410397.2026.2643037)
Supplement: Supplemental File. Search strategies and included African countries. [file ZRHM_A_2643037_SM3932.docx]

Supplemental File 1. Search Strategies

**1. Multi-database search strategies**

| **Database** | **Platform** | **Update** | **Date searched** |
| --- | --- | --- | --- |
| MEDLINE | Ovid | Ovid MEDLINE(R) ALL <1946 to October 26, 2023> | 2023-10-27 |
| Embase | Ovid | Embase Classic+Embase 1947 to 2023 October 26 | 2023-10-27 |
| PsycInfo | Ovid | APA PsycInfo <1806 to October Week 3 2023> | 2023-10-27 |
| CINAHL | EBSCO | 1937 to October 27, 2023 | 2023-10-27 |
| Global Health | EBSCO | update interval not public for this database | 2023-10-27 |

MEDLINE (Ovid)

| 1 | exp contraception/ |
| --- | --- |
| 2 | Reproductive behavior/ or Contraception behavior/ |
| 3 | exp Contraceptive agents/ |
| 4 | exp Contraceptive Devices/ |
| 5 | (condom* or (OC adj pill) or (intrauterine system or intra-uterine system or IUS or intrauterine device* or intra-uterine device* or IUD*) or (vasectomy or sterilisation or sterilization or (tubal adj ligation)) or ((vaginal adj ring) or ((abstain or abstinen*) adj2 (sex* or intercourse)) or lactational amenorr*)).ti,ab,kf. |
| 6 | (contracept* or family planning or (birth adj (control or regulat* or spacing)) or planned parenthood or ((population or fertility) adj (regulat* or control))).ti,ab,kf. |
| 7 | Pregnancy in Adolescence/ |
| 8 | (pregnan* adj2 (adolescen* or teen* or schoolchild* or youth* or preteen* or pre-teen*)).ti,ab,kf. |
| 9 | Pregnancy, unplanned/ or Pregnancy, unwanted/ |
| 10 | (pregnan* adj3 (prevent* or interrupt* or unplanned or unwanted or mistimed)).ti,ab,kf. |
| 11 | exp Sexually Transmitted Diseases/ |
| 12 | (sexually transmi* or STI or STIs or STD or STDs).ti,ab,kf. |
| 13 | (hiv or hiv-1* or hiv-2* or hiv1 or hiv2 or human immunodeficiency virus or human immunedeficiency virus or human immuno-deficiency virus or human immune-deficiency virus or (human immun* and deficiency virus) or acquired immunodeficiency syndrome or acquired immunedeficiency syndrome or acquired immuno-deficiency syndrome or acquired immune-deficiency syndrome or (acquired immun* and deficiency syndrome)).ti,ab,kf. |
| 14 | exp Anti-HIV Agents/ or Antiretroviral Therapy, Highly Active/ |
| 15 | (antiretroviral* or anti-retroviral* or ARV*).ti,ab,kf. |
| 16 | Papillomavirus Vaccines/ |
| 17 | (Chancroid or chlamydia or gonorrhea or syphilis or (hpv or papilloma virus* or papillomavirus*) or (genital* adj herpes)).ti,ab,kf. |
| 18 | Domestic Violence/ or Spouse Abuse/ or Intimate Partner Violence/ or Rape/ or Gender-Based Violence/ |
| 19 | (((sexual or domestic or spous* or intimate partner or gender-based) adj3 (violen* or abus*)) or rape).ti,ab,kf. |
| 20 | Puberty/ |
| 21 | (pubert* or pubescen*).ti,ab,kf. |
| 22 | Menstruation/ |
| 23 | (menstruat* or menstrual*).ti,ab,kf. |
| 24 | Abortion, Legal/ |
| 25 | Abortion, Induced/ |
| 26 | (abort* or miscarr* or (pregnan* adj2 terminat*)).ti,ab,kf. |
| 27 | Sexual behavior/ or Sex work/ or Safe sex/ or Unsafe sex/ |
| 28 | (sex* adj3 (protected or unprotected or safe or unsafe or risk* or behavio*)).ti,ab,kf. |
| 29 | sexual health/ |
| 30 | (sexual adj3 health).ti,ab,kf. |
| 31 | Reproductive Health/ |
| 32 | (reproductive adj3 health).ti,ab,kf. |
| 33 | sex education/ |
| 34 | (sex* adj (education* or "health promot*")).ti,ab,kf. |
| 35 | or/1-34 [Concept 1: SHRHR topics] |
| 36 | Adolescent/ or Young adult/ or Child/ |
| 37 | (child* or kid* or preteen* or pre-teen* or teen* or youth* or adolescen* or juvenile* or (young adj2 (adult* or person* or individual* or people* or population* or man or men or wom#n)) or youngster* or highschool*).ti,ab,kf. |
| 38 | or/36-37 [Concept 2: adolescent] |
| 39 | exp "Africa South of the Sahara"/ or Mauritius/ |
| 40 | (Angola or Botswana or Eswatini or Swaziland or Lesotho or Malawi or Mozambique or Namibia or South Africa or Zambia or Zimbabwe).tw,kf. |
| 41 | (Comoros or Djibouti or Eritrea or Ethiopia or Kenya or Madagascar or Mauritius or Rwanda or Seychelles or Somalia or South Sudan or Sudan or Tanzania or Uganda).tw,kf. |
| 42 | (Benin or Burkina Faso or Cabo Verde or Cote d'Ivoire or Ivory Coast or Gambia or Ghana or Guinea-Bissau or Guinea or Liberia or Mali or Niger or Nigeria or Senegal or Sierra Leone or Togo).tw,kf. |
| 43 | (Burundi or Cameroon or Central African Republic or Chad or Congo or Congo Republic or DR Congo or Democratic Republic of Congo or Equatorial Guinea or Gabon or "Sao Tome and Principe").tw,kf. |
| 44 | or/39-43 [Concept 3: sub-Saharan Africa] |
| 45 | exp Peer Group/ |
| 46 | (peer* adj3 (led or coach* or deliver* or based or promot* or counsel* or mentor* or engage*)).ti,ab,kf. |
| 47 | participatory.tw,kf. |
| 48 | ((youth* or teen* or child* or adolescent* or (young adj2 women) or girls) adj3 (design or promot* or collab* or participat* or engag* or consult*)).tw,kf. |
| 49 | ((youth* or teen* or child* or adolescent*) adj3 friendl*).tw,kf. |
| 50 | Health Knowledge, Attitudes, Practice/ |
| 51 | ((youth* or teen* or child* or adolescent* or (young adj2 women) or girls) adj3 (views or preferences or behavio?r or attitude* or perspective* or experiences or needs or feedback)).tw,kf. |
| 52 | or/45-51 [Concept 4: youth-involvement] |
| 53 | Program evaluation/ or Program development/ |
| 54 | (program? or programme? or service? or intervention? or project? or initiative?).ti,kf. |
| 55 | ((health or healthcare) adj15 (program? or programme? or service? or intervention? or project? or initiative?)).ab. |
| 56 | or/53-55 [Concept 5: interventions] |
| 57 | 35 and 38 and 44 and 52 and 56 |

Embase (Ovid)

| 1 | exp contraception/ or birth control/ |
| --- | --- |
| 2 | exp contraceptive behavior/ or reproductive behavior/ |
| 3 | exp contraceptive agent/ |
| 4 | exp Contraceptive Device/ |
| 5 | (condom* or (OC adj pill) or (intrauterine system or intra-uterine system or IUS or intrauterine device* or intra-uterine device* or IUD*) or (vasectomy or sterilisation or sterilization or (tubal adj ligation)) or ((vaginal adj ring) or ((abstain or abstinen*) adj2 (sex* or intercourse)) or lactational amenorr*)).ti,ab,kf. |
| 6 | (contracept* or family planning or (birth adj (control or regulat* or spacing)) or planned parenthood or ((population or fertility) adj (regulat* or control))).ti,ab,kf. |
| 7 | adolescent pregnancy/ |
| 8 | (pregnan* adj2 (adolescen* or teen* or schoolchild* or youth* or preteen* or pre-teen*)).ti,ab,kf. |
| 9 | unplanned pregnancy/ or unwanted pregnancy/ |
| 10 | (pregnan* adj3 (prevent* or interrupt* or unplanned or unwanted or mistimed)).ti,ab,kf. |
| 11 | exp sexually transmitted disease/ or exp Human immunodeficiency virus infection/ or exp papillomavirus infection/ or exp chlamydia infection/ |
| 12 | (sexually transmi* or STI or STIs or STD or STDs).ti,ab,kf. |
| 13 | (hiv or hiv-1* or hiv-2* or hiv1 or hiv2 or human immunodeficiency virus or human immunedeficiency virus or human immuno-deficiency virus or human immune-deficiency virus or (human immun* and deficiency virus) or acquired immunodeficiency syndrome or acquired immunedeficiency syndrome or acquired immuno-deficiency syndrome or acquired immune-deficiency syndrome or (acquired immun* and deficiency syndrome)).ti,ab,kf. |
| 14 | anti human immunodeficiency virus agent/ or human immunodeficiency virus fusion inhibitor/ or human immunodeficiency virus proteinase inhibitor/ or highly active antiretroviral therapy/ |
| 15 | (antiretroviral* or anti-retroviral* or ARV*).ti,ab,kf. |
| 16 | Human papilloma virus vaccine/ |
| 17 | (Chancroid or chlamydia or gonorrhea or syphilis or (hpv or papilloma virus* or papillomavirus*) or (genital* adj herpes)).ti,ab,kf. |
| 18 | domestic violence/ or exp partner violence/ or exp sexual violence/ or gender-based violence/ |
| 19 | (((sexual or domestic or spous* or intimate partner or gender-based) adj3 (violen* or abus*)) or rape).ti,ab,kf. |
| 20 | exp puberty/ |
| 21 | (pubert* or pubescen*).ti,ab,kf. |
| 22 | Menstruation/ |
| 23 | (menstruat* or menstrual*).ti,ab,kf. |
| 24 | legal abortion/ |
| 25 | induced abortion/ or medical abortion/ |
| 26 | (abort* or miscarr* or (pregnan* adj2 terminat*)).ti,ab,kf. |
| 27 | sexual behavior/ or adolescent sexual behavior/ or exp casual sex/ or concurrent sexual partnership/ or exp safe sex/ or exp unsafe sex/ or prostitution/ |
| 28 | (sex* adj3 (protected or unprotected or safe or unsafe or risk* or behavio*)).ti,ab,kf. |
| 29 | exp sexual health/ |
| 30 | (sexual adj3 health).ti,ab,kf. |
| 31 | Reproductive Health/ |
| 32 | (reproductive adj3 health).ti,ab,kf. |
| 33 | sex education/ |
| 34 | (sex* adj (education* or "health promot*")).ti,ab,kf. |
| 35 | or/1-34 [Concept 1: SHRHR topics] |
| 36 | exp adolescent/ or exp adolescence/ or young adult/ or child/ or girl/ or School child/ |
| 37 | (child* or kid* or preteen* or pre-teen* or teen* or youth* or adolescen* or juvenile* or (young adj2 (adult* or person* or individual* or people* or population* or man or men or wom#n)) or youngster* or highschool*).ti,ab,kf. |
| 38 | or/36-37 [Concept 2: adolescent] |
| 39 | exp "Africa South of the Sahara"/ or Mauritius/ |
| 40 | (Angola or Botswana or Eswatini or Swaziland or Lesotho or Malawi or Mozambique or Namibia or South Africa or Zambia or Zimbabwe).tw,kf. |
| 41 | (Comoros or Djibouti or Eritrea or Ethiopia or Kenya or Madagascar or Mauritius or Rwanda or Seychelles or Somalia or South Sudan or Sudan or Tanzania or Uganda).tw,kf. |
| 42 | (Benin or Burkina Faso or Cabo Verde or Cote d'Ivoire or Ivory Coast or Gambia or Ghana or Guinea-Bissau or Guinea or Liberia or Mali or Niger or Nigeria or Senegal or Sierra Leone or Togo).tw,kf. |
| 43 | (Burundi or Cameroon or Central African Republic or Chad or Congo or Congo Republic or DR Congo or Democratic Republic of Congo or Equatorial Guinea or Gabon or "Sao Tome and Principe").tw,kf. |
| 44 | or/39-43 [Concept 3: sub-Saharan Africa] |
| 45 | exp peer group/ or peer counseling/ |
| 46 | (peer* adj3 (led or coach* or deliver* or based or promot* or counsel* or mentor* or engage*)).ti,ab,kf. |
| 47 | participatory.tw,kf. |
| 48 | ((youth* or teen* or child* or adolescent* or (young adj2 women) or girls) adj3 (design or promot* or collab* or participat* or engag* or consult*)).tw,kf. |
| 49 | ((youth* or teen* or child* or adolescent*) adj3 friendl*).tw,kf. |
| 50 | attitude to health/ |
| 51 | ((youth* or teen* or child* or adolescent* or (young adj2 women) or girls) adj3 (views or preferences or behavio?r or attitude* or perspective* or experiences or needs or feedback)).tw,kf. |
| 52 | or/45-51 [Concept 4: youth-involvement] |
| 53 | Program evaluation/ or Program development/ |
| 54 | (program? or programme? or service? or intervention? or project? or initiative?).ti,kf. |
| 55 | ((health or healthcare) adj15 (program? or programme? or service? or intervention? or project? or initiative?)).ab. |
| 56 | or/53-55 [Concept 5: interventions] |
| 57 | 35 and 38 and 44 and 52 and 56 |

APA PsycInfo (Ovid)

| 1 | birth control/ |
| --- | --- |
| 2 | family planning/ |
| 3 | exp contraceptive devices/ |
| 4 | (condom* or (OC adj pill) or (intrauterine system or intra-uterine system or IUS or intrauterine device* or intra-uterine device* or IUD*) or (vasectomy or sterilisation or sterilization or (tubal adj ligation)) or ((vaginal adj ring) or ((abstain or abstinen*) adj2 (sex* or intercourse)) or lactational amenorr*)).ti,ab. |
| 5 | (contracept* or family planning or (birth adj (control or regulat* or spacing)) or planned parenthood or ((population or fertility) adj (regulat* or control))).ti,ab. |
| 6 | Adolescent Pregnancy/ |
| 7 | (pregnan* adj2 (adolescen* or teen* or schoolchild* or youth* or preteen* or pre-teen*)).ti,ab. |
| 8 | (pregnan* adj3 (prevent* or interrupt* or unplanned or unwanted or mistimed)).ti,ab. |
| 9 | exp Sexually Transmitted Diseases/ or Human Papillomavirus/ |
| 10 | (sexually transmi* or STI or STIs or STD or STDs).ti,ab. |
| 11 | (hiv or hiv-1* or hiv-2* or hiv1 or hiv2 or human immunodeficiency virus or human immunedeficiency virus or human immuno-deficiency virus or human immune-deficiency virus or (human immun* and deficiency virus) or acquired immunodeficiency syndrome or acquired immunedeficiency syndrome or acquired immuno-deficiency syndrome or acquired immune-deficiency syndrome or (acquired immun* and deficiency syndrome)).ti,ab. |
| 12 | anti human immunodeficiency virus agent/ or human immunodeficiency virus fusion inhibitor/ or human immunodeficiency virus proteinase inhibitor/ or highly active antiretroviral therapy.mp. [mp=title, abstract, heading word, table of contents, key concepts, original title, tests & measures, mesh word] |
| 13 | (antiretroviral* or anti-retroviral* or ARV*).ti,ab. |
| 14 | (Chancroid or chlamydia or gonorrhea or syphilis or (hpv or papilloma virus* or papillomavirus*) or (genital* adj herpes)).ti,ab. |
| 15 | Domestic violence/ or exp Gender violence/ or battered females/ |
| 16 | (((sexual or domestic or spous* or intimate partner) adj3 (violen* or abus*)) or rape).ti,ab. |
| 17 | puberty/ |
| 18 | (pubert* or pubescen*).ti,ab. |
| 19 | exp menstruation/ |
| 20 | (menstruat* or menstrual*).ti,ab. |
| 21 | Induced abortion/ |
| 22 | Abortion (Attitudes Toward)/ |
| 23 | (abort* or miscarr* or (pregnan* adj2 terminat*)).ti,ab. |
| 24 | (sex* adj3 (protected or unprotected or safe or unsafe or risk* or behavio*)).ti,ab. |
| 25 | sexual health/ |
| 26 | (sexual adj3 health).ti,ab. |
| 27 | Reproductive Health/ |
| 28 | (reproductive adj3 health).ti,ab. |
| 29 | sex education/ |
| 30 | (sex* adj (education* or "health promot*")).ti,ab. |
| 31 | or/1-30 |
| 32 | child health/ or adolescent health/ |
| 33 | (child* or kid* or preteen* or pre-teen* or teen* or youth* or adolescen* or juvenile* or (young adj2 (adult* or person* or individual* or people* or population* or man or men or wom#n)) or youngster* or highschool*).ti,ab. |
| 34 | or/32-33 |
| 35 | (Angola or Botswana or Eswatini or Swaziland or Lesotho or Malawi or Mozambique or Namibia or South Africa or Zambia or Zimbabwe).ti,ab. |
| 36 | (Comoros or Djibouti or Eritrea or Ethiopia or Kenya or Madagascar or Mauritius or Rwanda or Seychelles or Somalia or South Sudan or Sudan or Tanzania or Uganda).ti,ab. |
| 37 | (Benin or Burkina Faso or Cabo Verde or Cote d'Ivoire or Ivory Coast or Gambia or Ghana or Guinea-Bissau or Guinea or Liberia or Mali or Niger or Nigeria or Senegal or Sierra Leone or Togo).ti,ab. |
| 38 | (Burundi or Cameroon or Central African Republic or Chad or Congo or Congo Republic or DR Congo or Democratic Republic of Congo or Equatorial Guinea or Gabon or "Sao Tome and Principe").ti,ab. |
| 39 | or/35-38 |
| 40 | Peers/ |
| 41 | (peer* adj3 (led or coach* or deliver* or based or promot* or counsel* or mentor* or engage*)).ti,ab. |
| 42 | participatory.ti,ab. |
| 43 | ((youth* or teen* or child* or adolescent* or (young adj2 women) or girls) adj3 (design or promot* or collab* or participat* or engag* or consult*)).ti,ab. |
| 44 | ((youth* or teen* or child* or adolescent*) adj3 friendl*).ti,ab. |
| 45 | health knowledge/ or exp Health Attitudes/ or exp Health behavior/ |
| 46 | ((youth* or teen* or child* or adolescent* or (young adj2 women) or girls) adj3 (views or preferences or behavio?r or attitude* or perspective* or experiences or needs or feedback)).ti,ab. |
| 47 | or/40-46 |
| 48 | Program Development/ or Program Evaluation/ or Mental Health Program Evaluation/ |
| 49 | (program? or programme? or service? or intervention? or project? or initiative?).ti. |
| 50 | ((health or healthcare) adj15 (program? or programme? or service? or intervention? or project? or initiative?)).ab. |
| 51 | 48 or 49 or 50 |
| 52 | 31 and 34 and 39 and 47 and 51 |
| 53 | ("Chain Peer Referral Approach for HIV Testing Among Adolescents in Kisumu County, Kenya" or "Effects of a Peer-Led Intervention on HIV Care Continuum Outcomes Among Contacts of Children, Adolescents, and Young Adults Living With HIV in Zimbabwe" or "Does making clinic-based reproductive health services more youth-friendly increase service use by adolescents? Evidence from Lusaka, Zambia" or "Adolescents' views of and preferences for sexual and reproductive health services in Burkina Faso, Ghana, Malawi and Uganda" or "A Participatory Comic Book Workshop to Improve Youth-Friendly Post-Rape Care in a Humanitarian Context in Uganda: A Case Study" or "Experiences of antenatal care among pregnant adolescents at Kanyama and Matero clinics in Lusaka district, Zambia" or ("The feasibility and acceptability of Project POWER: a mindfulness- infused, cognitive-behavioral group intervention to address mental and sexual health needs of young pregnant women in Liberia" or "Promoting adolescent health through integrated human papillomavirus vaccination programs: The experience of Togo" or "Quality assessment in primary health care: Adolescent and Youth Friendly Service, a Mozambican case study")).ti. |
| 54 | 52 or 53 |

CINAHL (Ebsco)

No expanders or equivalent subjects applied

| # | Query |
| --- | --- |
| S75 | S52 AND S53 AND S69 AND S70 AND S74 |
| S74 | S71 OR S72 OR S73 |
| S73 | ab((health or healthcare) N14 (program# or programme# or service# or intervention# or project# or initiative#)) |
| S72 | TI(program# or programme# or service# or intervention# or project# or initiative#) |
| S71 | (MH "Program Development") OR (MH "Program Planning") OR (MH "Program Evaluation") OR (MH "Program Implementation") OR (MH "Hospital Programs") |
| S70 | S57 OR S58 OR S59 OR S60 OR S61 OR S62 OR S63 OR S64 OR S65 OR S66 OR S67 OR S68 |
| S69 | S54 OR S55 OR S56 |
| S68 | AB((youth* or teen* or child* or adolescent* or (young N1 women) or girls) N2 (views or preferences or behavio?r or attitude* or perspective* or experiences or needs or feedback)) |
| S67 | TI((youth* or teen* or child* or adolescent* or (young N1 women) or girls) N2 (views or preferences or behavio?r or attitude* or perspective* or experiences or needs or feedback)) |
| S66 | (MH "Health Knowledge") OR (MH "Attitude to Health") |
| S65 | AB((youth* or teen* or child* or adolescent*) N2 friendl*) |
| S64 | TI((youth* or teen* or child* or adolescent*) N2 friendl*) |
| S63 | AB((youth* or teen* or child* or adolescent* or (young N1 women) or girls) N2 (design or promot* or collab* or participat* or engag* or consult*)) |
| S62 | TI((youth* or teen* or child* or adolescent* or (young N1 women) or girls) N2 (design or promot* or collab* or participat* or engag* or consult*)) |
| S61 | AB(participatory) |
| S60 | TI(participatory) |
| S59 | AB(peer* N2 (led or coach* or deliver* or based or promot* or counsel* or mentor* or engage*)) |
| S58 | TI(peer* N2 (led or coach* or deliver* or based or promot* or counsel* or mentor* or engage*)) |
| S57 | (MH "Peer Group") OR (MH "Peer Counseling") OR (MH "Peer Assistance Programs") |
| S56 | (( TI(Benin or "Burkina Faso" or "Cabo Verde" or "Cote d'Ivoire" or "Ivory Coast" or Gambia or Ghana or "Guinea-Bissau" or Guinea or Liberia or Mali or Niger or Nigeria or Senegal or "Sierra Leone" or Togo) ) OR ( AB(Benin or "Burkina Faso" or "Cabo Verde" or "Cote d'Ivoire" or "Ivory Coast" or Gambia or Ghana or "Guinea-Bissau" or Guinea or Liberia or Mali or Niger or Nigeria or Senegal or "Sierra Leone" or Togo) ) OR ( TI(Burundi or Cameroon or "Central African Republic" or Chad or Congo or Congo Republic or "DR Congo" or "Democratic Republic of Congo" or "Equatorial Guinea" or Gabon or "Sao Tome and Principe") ) OR ( AB(Burundi or Cameroon or "Central African Republic" or Chad or Congo or Congo Republic or "DR Congo" or "Democratic Republic of Congo" or "Equatorial Guinea" or Gabon or "Sao Tome and Principe") )) AND (S54 OR S55) |
| S55 | ( TI(Benin or "Burkina Faso" or "Cabo Verde" or "Cote d'Ivoire" or "Ivory Coast" or Gambia or Ghana or "Guinea-Bissau" or Guinea or Liberia or Mali or Niger or Nigeria or Senegal or "Sierra Leone" or Togo) ) OR ( AB(Benin or "Burkina Faso" or "Cabo Verde" or "Cote d'Ivoire" or "Ivory Coast" or Gambia or Ghana or "Guinea-Bissau" or Guinea or Liberia or Mali or Niger or Nigeria or Senegal or "Sierra Leone" or Togo) ) OR ( TI(Burundi or Cameroon or "Central African Republic" or Chad or Congo or Congo Republic or "DR Congo" or "Democratic Republic of Congo" or "Equatorial Guinea" or Gabon or "Sao Tome and Principe") ) OR ( AB(Burundi or Cameroon or "Central African Republic" or Chad or Congo or Congo Republic or "DR Congo" or "Democratic Republic of Congo" or "Equatorial Guinea" or Gabon or "Sao Tome and Principe") ) |
| S54 | (MH "Africa South of the Sahara"+) OR ( TI(Angola or Botswana or Eswatini or Swaziland or Lesotho or Malawi or Mozambique or Namibia or "South Africa" or Zambia or Zimbabwe) ) OR ( TI(Comoros or Djibouti or Eritrea or Ethiopia or Kenya or Madagascar or Mauritius or Rwanda or Seychelles or Somalia or South Sudan or Sudan or Tanzania or Uganda) ) OR ( AB(Angola or Botswana or Eswatini or Swaziland or Lesotho or Malawi or Mozambique or Namibia or "South Africa" or Zambia or Zimbabwe) ) OR ( AB(Comoros or Djibouti or Eritrea or Ethiopia or Kenya or Madagascar or Mauritius or Rwanda or Seychelles or Somalia or South Sudan or Sudan or Tanzania or Uganda) ) |
| S53 | ( (MH "Adolescence") OR (MH "Young Adult") OR (MH "Child") ) OR ( TI(child* or kid* or preteen* or "pre-teen*" or teen* or youth* or adolescen* or juvenile* or (young N1 (adult* or person* or individual* or people* or population* or man or men or wom?n)) or youngster* or highschool*) ) OR ( AB(child* or kid* or preteen* or "pre-teen*" or teen* or youth* or adolescen* or juvenile* or (young N1 (adult* or person* or individual* or people* or population* or man or men or wom?n)) or youngster* or highschool*) ) |
| S52 | S1 OR S2 OR S3 OR S4 OR S5 OR S6 OR S7 OR S8 OR S9 OR S10 OR S11 OR S12 OR S13 OR S14 OR S15 OR S16 OR S17 OR S18 OR S19 OR S20 OR S21 OR S22 OR S23 OR S24 OR S25 OR S26 OR S27 OR S28 OR S29 OR S30 OR S31 OR S32 OR S33 OR S34 OR S35 OR S36 OR S37 OR S38 OR S39 OR S40 OR S41 OR S42 OR S43 OR S44 OR S45 OR S46 OR S47 OR S48 OR S49 OR S50 OR S51 |
| S51 | AB(sex* N0 (education* or "health promot*")) |
| S50 | TI(sex* N0 (education* or "health promot*")) |
| S49 | (MH "Sex Education") |
| S48 | TI(reproductive N2 health) |
| S47 | (MH "Reproductive Health") |
| S46 | AB(sexual N2 health) |
| S45 | TI(sexual N2 health) |
| S44 | (MH "Sexual Health") |
| S43 | AB(sex* N2 (protected or unprotected or safe or unsafe or risk* or behavio*)) |
| S42 | TI(sex* N2 (protected or unprotected or safe or unsafe or risk* or behavio*)) |
| S41 | (MH "Sexual Behavior") OR (MH "Unsafe Sex") OR (MH "Safe Sex") OR (MH "Sex Work"+) |
| S40 | AB(abort* or miscarr* or (pregnan* N1 terminat*)) |
| S39 | TI(abort* or miscarr* or (pregnan* N1 terminat*)) |
| S38 | (MH "Attitude to Abortion") |
| S37 | (MH "Abortion, Induced") |
| S36 | AB(menstruat* or menstrual*) |
| S35 | TI(menstruat* or menstrual*) |
| S34 | (MH "Menstruation") |
| S33 | AB(pubert* or pubescen*) |
| S32 | TI(pubert* or pubescen*) |
| S31 | (MH "Puberty+") |
| S30 | AB(((sexual or domestic or spous* or "intimate partner" or "gender-based") N2 (violen* or abus*)) or rape) |
| S29 | TI(((sexual or domestic or spous* or "intimate partner" or "gender-based") N2 (violen* or abus*)) or rape) |
| S28 | (MH "Intimate Partner Violence") OR (MH "Domestic Violence") OR (MH "Gender-Based Violence") OR (MH "Intimate Partner Violence") OR (MH "Domestic Violence") OR (MH "Gender-Based Violence") |
| S27 | AB(Chancroid or chlamydia or gonorrhea or syphilis or (hpv or "papilloma virus*" or papillomavirus*) or "genital herpes" or "herpes genitalis")) |
| S26 | TI(Chancroid or chlamydia or gonorrhea or syphilis or (hpv or "papilloma virus*" or papillomavirus*) or "genital herpes" or "herpes genitalis")) |
| S25 | (MH "Papillomavirus Vaccine") |
| S24 | AB(antiretroviral* or "anti-retroviral*" or ARV*) |
| S23 | TI(antiretroviral* or "anti-retroviral*" or ARV*) |
| S22 | (MH "Antiretroviral Therapy, Highly Active") OR (MH "Anti-HIV Agents+") |
| S21 | AB(hiv or hiv-1* or hiv-2* or hiv1 or hiv2 or "human immunodeficiency virus" or "human immunedeficiency virus" or "human immuno-deficiency virus" or "human immune-deficiency virus" or ("human immun*" and "deficiency virus") or "acquired immunodeficiency syndrome" or "acquired immunedeficiency syndrome" or "acquired immuno-deficiency syndrome" or "acquired immune-deficiency syndrome" or ("acquired immun*" and "deficiency syndrome")) |
| S20 | TI(hiv or hiv-1* or hiv-2* or hiv1 or hiv2 or "human immunodeficiency virus" or "human immunedeficiency virus" or "human immuno-deficiency virus" or "human immune-deficiency virus" or ("human immun*" and "deficiency virus") or "acquired immunodeficiency syndrome" or "acquired immunedeficiency syndrome" or "acquired immuno-deficiency syndrome" or "acquired immune-deficiency syndrome" or ("acquired immun*" and "deficiency syndrome")) |
| S19 | AB("sexually transmi*" or STI or STIs or STD or STDs) |
| S18 | TI("sexually transmi*" or STI or STIs or STD or STDs) |
| S17 | (MH "Sexually Transmitted Diseases+") |
| S16 | AB(pregnan* N2 (prevent* or interrupt* or unplanned or unwanted or mistimed)) |
| S15 | TI(pregnan* N2 (prevent* or interrupt* or unplanned or unwanted or mistimed)) |
| S14 | (MH "Pregnancy, Unplanned") OR (MH "Pregnancy, Unwanted") |
| S13 | (MH "Pregnancy, Unplanned") OR (MH "Pregnancy, Unwanted") |
| S12 | (MH "Pregnancy, Unplanned") OR (MH "Pregnancy, Unwanted") |
| S11 | AB(pregnan* N1 (adolescen* or teen* or schoolchild* or youth* or preteen* or "pre-teen*")) |
| S10 | TI(pregnan* N1 (adolescen* or teen* or schoolchild* or youth* or preteen* or "pre-teen*")) |
| S9 | (MH "Pregnancy in Adolescence+") |
| S8 | AB(contracept* or "family planning" or (birth N0 (control or regulat* or spacing)) or "planned parenthood" or ((population or fertility) N0 (regulat* or control))) |
| S7 | TI(contracept* or "family planning" or (birth N0 (control or regulat* or spacing)) or "planned parenthood" or ((population or fertility) N0 (regulat* or control))) |
| S6 | AB(condom* or (OC adj pill) or ("intrauterine system" or "intra-uterine system" or IUS or "intrauterine device*" or "intra-uterine device*" or IUD*) or (vasectomy or sterilisation or sterilization or (tubal N0 ligation)) or ((vaginal N0 ring) or ((abstain or abstinen*) N1 (sex* or intercourse)) or "lactational amenorr*")) |
| S5 | TI(condom* or (OC adj pill) or ("intrauterine system" or "intra-uterine system" or IUS or "intrauterine device*" or "intra-uterine device*" or IUD*) or (vasectomy or sterilisation or sterilization or (tubal N0 ligation)) or ((vaginal N0 ring) or ((abstain or abstinen*) N1 (sex* or intercourse)) or "lactational amenorr*")) |
| S4 | (MH "Contraceptive Devices+") |
| S3 | (MH "Contraceptive Agents+") |
| S2 | (MH "Family Planning+") OR (MH "Reproductive Behavior") |
| S1 | (MH "Contraception+") |

Global Health (Ebsco)

No expanders or equivalent subjects applied

| S52 | S30 AND S31 AND S36 AND S46 AND S51 |
| --- | --- |
| S51 | S47 OR S48 OR S49 OR S50 |
| S50 | (ab((health or healthcare) N14 (program# or programme# or service# or intervention# or project# or initiative#))) |
| S49 | ab((health or healthcare) N14 (program# or programme# or service# or intervention# or project# or initiative#)) |
| S48 | TI(program# or programme# or service# or intervention# or project# or initiative#) |
| S47 | DE "health programs" OR DE "program development" OR DE "program effectiveness" OR DE "program evaluation" |
| S46 | S37 OR S38 OR S39 OR S40 OR S41 OR S42 OR S43 OR S44 OR S45 |
| S45 | TI((youth* or teen* or child* or adolescent* or (young N1 women) or girls) N2 (views or preferences or behavio?r or attitude* or perspective* or experiences or needs or feedback)) |
| S44 | TI((youth* or teen* or child* or adolescent* or (young N1 women) or girls) N2 (views or preferences or behavio?r or attitude* or perspective* or experiences or needs or feedback)) |
| S43 | AB((youth* or teen* or child* or adolescent*) N2 friendl*) |
| S42 | TI((youth* or teen* or child* or adolescent*) N2 friendl*) |
| S41 | AB((youth* or teen* or child* or adolescent* or (young N1 women) or girls) N2 (design or promot* or collab* or participat* or engag* or consult*)) |
| S40 | TI((youth* or teen* or child* or adolescent* or (young N1 women) or girls) N2 (design or promot* or collab* or participat* or engag* or consult*)) |
| S39 | TI(participatory) OR AB(participatory) |
| S38 | TI(peer* N2 (led or coach* or deliver* or based or promot* or counsel* or mentor* or engage*)) |
| S37 | DE "peer relationships" OR DE "peer tutoring" |
| S36 | (DE "Africa South of Sahara" OR DE "Central Africa" OR DE "East Africa" OR DE "Sahel" OR DE "Southern Africa" OR DE "West Africa") OR (S32 OR S33 OR S34 OR S35) |
| S35 | DE "Africa South of Sahara" OR DE "Central Africa" OR DE "East Africa" OR DE "Sahel" OR DE "Southern Africa" OR DE "West Africa" |
| S34 | (( TI(Benin or "Burkina Faso" or "Cabo Verde" or "Cote d'Ivoire" or "Ivory Coast" or Gambia or Ghana or "Guinea-Bissau" or Guinea or Liberia or Mali or Niger or Nigeria or Senegal or "Sierra Leone" or Togo) ) OR ( AB(Benin or "Burkina Faso" or "Cabo Verde" or "Cote d'Ivoire" or "Ivory Coast" or Gambia or Ghana or "Guinea-Bissau" or Guinea or Liberia or Mali or Niger or Nigeria or Senegal or "Sierra Leone" or Togo) ) OR ( TI(Burundi or Cameroon or "Central African Republic" or Chad or Congo or Congo Republic or "DR Congo" or "Democratic Republic of Congo" or "Equatorial Guinea" or Gabon or "Sao Tome and Principe") ) OR ( AB(Burundi or Cameroon or "Central African Republic" or Chad or Congo or Congo Republic or "DR Congo" or "Democratic Republic of Congo" or "Equatorial Guinea" or Gabon or "Sao Tome and Principe") )) AND (S32 OR S33) |
| S33 | ( TI(Benin or "Burkina Faso" or "Cabo Verde" or "Cote d'Ivoire" or "Ivory Coast" or Gambia or Ghana or "Guinea-Bissau" or Guinea or Liberia or Mali or Niger or Nigeria or Senegal or "Sierra Leone" or Togo) ) OR ( AB(Benin or "Burkina Faso" or "Cabo Verde" or "Cote d'Ivoire" or "Ivory Coast" or Gambia or Ghana or "Guinea-Bissau" or Guinea or Liberia or Mali or Niger or Nigeria or Senegal or "Sierra Leone" or Togo) ) OR ( TI(Burundi or Cameroon or "Central African Republic" or Chad or Congo or Congo Republic or "DR Congo" or "Democratic Republic of Congo" or "Equatorial Guinea" or Gabon or "Sao Tome and Principe") ) OR ( AB(Burundi or Cameroon or "Central African Republic" or Chad or Congo or Congo Republic or "DR Congo" or "Democratic Republic of Congo" or "Equatorial Guinea" or Gabon or "Sao Tome and Principe") ) |
| S32 | ( TI(Angola or Botswana or Eswatini or Swaziland or Lesotho or Malawi or Mozambique or Namibia or "South Africa" or Zambia or Zimbabwe) ) OR ( AB(Angola or Botswana or Eswatini or Swaziland or Lesotho or Malawi or Mozambique or Namibia or "South Africa" or Zambia or Zimbabwe) ) OR ( TI(Comoros or Djibouti or Eritrea or Ethiopia or Kenya or Madagascar or Mauritius or Rwanda or Seychelles or Somalia or South Sudan or Sudan or Tanzania or Uganda) ) OR ( AB(Comoros or Djibouti or Eritrea or Ethiopia or Kenya or Madagascar or Mauritius or Rwanda or Seychelles or Somalia or South Sudan or Sudan or Tanzania or Uganda) ) |
| S31 | ( DE "adolescents" OR DE "school children" OR DE "young adults" OR DE "youth" ) OR ( TI(child* or kid* or preteen* or "pre-teen*" or teen* or youth* or adolescen* or juvenile* or (young N1 (adult* or person* or individual* or people* or population* or man or men or wom?n)) or youngster* or highschool*) ) OR ( AB(child* or kid* or preteen* or "pre-teen*" or teen* or youth* or adolescen* or juvenile* or (young N1 (adult* or person* or individual* or people* or population* or man or men or wom?n)) or youngster* or highschool*) ) |
| S30 | S1 OR S2 OR S3 OR S4 OR S5 OR S6 OR S7 OR S8 OR S9 OR S10 OR S11 OR S12 OR S13 OR S14 OR S15 OR S16 OR S17 OR S18 OR S19 OR S20 OR S21 OR S22 OR S23 OR S24 OR S25 OR S26 OR S27 OR S28 OR S29 |
| S29 | AB(sex* N0 (education* or "health promot*")) |
| S28 | TI(sex* N0 (education* or "health promot*")) |
| S27 | (MH "Sex Education") |
| S26 | AB(reproductive N2 health) |
| S25 | DE "sex education" |
| S24 | AB(sexual N2 health) |
| S23 | TI(sexual N2 health) |
| S22 | ( TI(sex* N2 (protected or unprotected or safe or unsafe or risk* or behavio*)) ) OR ( AB(sex* N2 (protected or unprotected or safe or unsafe or risk* or behavio*)) ) |
| S21 | DE "safer sex" |
| S20 | ( TI(abort* or miscarr* or (pregnan* N1 terminat*)) ) OR ( AB(abort* or miscarr* or (pregnan* N1 terminat*)) ) |
| S19 | DE "abortion" OR DE "induced abortion" |
| S18 | ( TI(menstruat* or menstrual*) ) OR ( AB(menstruat* or menstrual*) ) |
| S17 | DE "menstruation" |
| S16 | ( DE "Puberty" OR DE "Menarche" ) OR ( TI(pubert* or pubescen*) ) OR ( AB(pubert* or pubescen*) ) |
| S15 | ( TI(((sexual or domestic or spous* or "intimate partner" or "gender-based") N2 (violen* or abus*)) or rape) ) AND ( AB(((sexual or domestic or spous* or "intimate partner" or "gender-based") N2 (violen* or abus*)) or rape) ) |
| S14 | DE "rape (trauma)" OR DE "abuse" OR DE "spouse abuse" |
| S13 | ( TI(Chancroid or chlamydia or gonorrhea or syphilis or (hpv or "papilloma virus*" or papillomavirus*) or "genital herpes" or "herpes genitalis")) ) OR ( AB(Chancroid or chlamydia or gonorrhea or syphilis or (hpv or "papilloma virus*" or papillomavirus*) or "genital herpes" or "herpes genitalis")) ) |
| S12 | ( TI(antiretroviral* or "anti-retroviral*" or ARV*) ) OR ( AB(antiretroviral* or "anti-retroviral*" or ARV*) ) |
| S11 | DE "antiretroviral agents" |
| S10 | ( TI(hiv or hiv-1* or hiv-2* or hiv1 or hiv2 or "human immunodeficiency virus" or "human immunedeficiency virus" or "human immuno-deficiency virus" or "human immune-deficiency virus" or ("human immun*" and "deficiency virus") or "acquired immunodeficiency syndrome" or "acquired immunedeficiency syndrome" or "acquired immuno-deficiency syndrome" or "acquired immune-deficiency syndrome" or ("acquired immun*" and "deficiency syndrome")) ) OR ( AB(hiv or hiv-1* or hiv-2* or hiv1 or hiv2 or "human immunodeficiency virus" or "human immunedeficiency virus" or "human immuno-deficiency virus" or "human immune-deficiency virus" or ("human immun*" and "deficiency virus") or "acquired immunodeficiency syndrome" or "acquired immunedeficiency syndrome" or "acquired immuno-deficiency syndrome" or "acquired immune-deficiency syndrome" or ("acquired immun*" and "deficiency syndrome")) ) |
| S9 | AB("sexually transmi*" or STI or STIs or STD or STDs) |
| S8 | TI("sexually transmi*" or STI or STIs or STD or STDs) |
| S7 | DE "sexually transmitted diseases" OR DE "chancroid" OR DE "gonorrhoea" OR DE "granuloma inguinale" OR DE "syphilis" OR DE "transmissible venereal tumour" OR DE "HIV infections" OR DE "HIV-1 infections" OR DE "HIV-2 infections" OR DE "human immunodeficiency viruses" OR DE "Human immunodeficiency virus 1" OR DE "Human immunodeficiency virus 2" |
| S6 | ( TI(pregnan* N2 (prevent* or interrupt* or unplanned or unwanted or mistimed)) ) OR ( AB(pregnan* N2 (prevent* or interrupt* or unplanned or unwanted or mistimed)) ) |
| S5 | ( TI(pregnan* N1 (adolescen* or teen* or schoolchild* or youth* or preteen* or "pre-teen*")) ) OR ( AB(pregnan* N1 (adolescen* or teen* or schoolchild* or youth* or preteen* or "pre-teen*")) ) |
| S4 | DE "pregnant adolescents" |
| S3 | ( TI(contracept* or "family planning" or (birth N0 (control or regulat* or spacing)) or "planned parenthood" or ((population or fertility) N0 (regulat* or control))) ) OR ( AB(contracept* or "family planning" or (birth N0 (control or regulat* or spacing)) or "planned parenthood" or ((population or fertility) N0 (regulat* or control))) ) |
| S2 | ( TI(condom* or (OC adj pill) or ("intrauterine system" or "intra-uterine system" or IUS or "intrauterine device*" or "intra-uterine device*" or IUD*) or (vasectomy or sterilisation or sterilization or (tubal N0 ligation)) or ((vaginal N0 ring) or ((abstain or abstinen*) N1 (sex* or intercourse)) or "lactational amenorr*")) ) OR ( AB(condom* or (OC adj pill) or ("intrauterine system" or "intra-uterine system" or IUS or "intrauterine device*" or "intra-uterine device*" or IUD*) or (vasectomy or sterilisation or sterilization or (tubal N0 ligation)) or ((vaginal N0 ring) or ((abstain or abstinen*) N1 (sex* or intercourse)) or "lactational amenorr*")) ) |
| S1 | ( DE "family planning" OR DE "contraception" ) OR DE "reproductive behaviour" OR ( DE "contraceptives" OR DE "condoms" OR DE "intrauterine devices" OR DE "oral contraceptives" ) |

**2. List of African countries**

To search for countries in Africa South of the Sahara, we used the African Union’s list of countries for the South, East, and West sections, listed below:

**South**

Angola

Botswana

Eswatini

Lesotho

Malawi

Mozambique

Namibia

South Africa

Zambia

Zimbabwe

**East**

Comoros

Djibouti

Eritrea

Ethiopia

Kenya

Madagascar

Mauritius

Rwanda

Seychelles

Somalia

South Sudan

Sudan

Tanzania

Uganda

**West**

Benin

Burkina Faso

Cabo Verde

Côte d'Ivoire

Gambia

Ghana

Guinea-Bissau

Guinea

Liberia

Mali

Niger

Nigeria

Senegal

Sierra Leone

Togo

**Central**

Burundi

Cameroon

Central African Republic

Chad

Congo Republic

DR Congo

Equatorial Guinea

Gabon

São Tomé and Príncipe
