# Supplementary material for: A systematic review of the effectiveness of participatory, health system-based interventions to improve the sexual and reproductive health and rights of adolescent girls and young women in Sub-Saharan Africa
Source: Sex Reprod Health Matters. 2026 Mar 18;33(1):2643037. doi: 10.1080/26410397.2026.2643037 (PMC13103997; doi:10.1080/26410397.2026.2643037)
Supplement: Supplemental Table 3. List of context articles for full text review. [file ZRHM_A_2643037_SM3931.docx]

| \| **Supplemental Table 3: List of 'Context' Articles for Full Text Review** \| \| \| \| \| \| \| --- \| --- \| --- \| --- \| --- \| --- \| \| **Covidence#** \| **Citation** \| **Country** \| **Participatory?** \| **Intervention** \| **Outcome** \| \| 5555 \| Steward WT, Sumitani J, Moran ME, Ratlhagana MJ, Morris JL, Isidoro L, Gilvydis JM, Tumbo J, Grignon J, Barnhart S, Lippman SA. Engaging HIV-positive clients in care: acceptability and mechanisms of action of a peer navigation program in South Africa. AIDS Care. 2018 Mar;30(3):330-337. doi: 10.1080/09540121.2017.1363362. Epub 2017 Aug 16. PMID: 28814110. \| South Africa \| Yes \| Peer navigation program \| Positive \| \| 58 \| Grace CS, Rakgadi GM. What's holding back youth-friendly health services in Blantyre, Malawi? A qualitative exploration. Afr J Reprod Health. 2023 Sep 30;27(9):57-64. doi: 10.29063/ajrh2023/v27i9.6. PMID: 37788347. \| Malawi \| Unclear \| N/A \| N/A \| \| 272 \| Mbengo, Fungai & Adama, Esther & Towell-Barnard, Amanda & Bhana, Arvin & Afrifa-Yamoah, Ebenezer & Zgambo, Maggie. (2022). Impact of you only live once: A resilience-based HIV prevention intervention to reduce risky sexual behaviour among youth in South Africa. Acta Psychologica. 230. 103757. 10.1016/j.actpsy.2022.103757. \| South Africa \| Yes \| YOLO curriculum \| Positive \| \| 403 \| Flanagan S, Gorstein A, Nicholson M, Bradish S, Amanyire D, Gidudu A, Aucur F, Twesigye J, Kyateka F, Balamaga S, Buttenheim A, Zimmerman E. Behavioural intervention for adolescent uptake of family planning: a randomized controlled trial, Uganda. Bull World Health Organ. 2021 Nov 1;99(11):795-804. doi: 10.2471/BLT.20.285339. Epub 2021 Sep 29. PMID: 34737472; PMCID: PMC8542266. \| Uganda \| No \| Peer-referral system \| Positive \| \| 52 \| Pleaner M, Scorgie F, Martin C, Butler V, Muhwava L, Mojapele M, Mullick S. Introduction and integration of PrEP and sexual and reproductive health services for young people: Health provider perspectives from South Africa. Front Reprod Health. 2023 Jan 9;4:1086558. doi: 10.3389/frph.2022.1086558. PMID: 36699145; PMCID: PMC9869154. \| South Africa \| No \| PrEP \| N/A \| \| 56 \| Merrill KG, Frimpong C, Burke VM, Abrams EA, Miti S, Mwansa JK, et al. (2023) “Project YES! has given me a task to reach undetectable”: Qualitative findings from a peer mentoring program for youth living with HIV in Zambia. PLoS ONE 18(10): e0292719. https://doi.org/10.1371/journal.pone.0292719 youth living with HIV in Zambia. \| Zambia \| No \| Peer-mentoring program \| Positive \| \| 59 \| Badejo O, Nöstlinger C, Wouters E, Laga M, Okonkwo P, Jwanle P, Van Belle S. Understanding why and how youth-friendly health services improve viral load suppression among adolescents and young people living with HIV in Nigeria: realist evaluation with qualitative comparative analysis. BMJ Glob Health. 2023 Sep;8(9):e012600. doi: 10.1136/bmjgh-2023-012600. PMID: 37748794; PMCID: PMC10533666. \| Nigeria \| Yes \| YFHS \| Positive \| \| 79 \| Zenebe GA, Ewunie TM, Belay MM, Abose AM. Quality of adolescent and youth-friendly sexual and reproductive health services and associated factors in Ethiopia: a systematic review and meta-analysis. Front Public Health. 2023 Jul 12;11:1191676. doi: 10.3389/fpubh.2023.1191676. PMID: 37501947; PMCID: PMC10369062. \| Ethiopia \| No \| Adolescent and youth-friendly sexual and reproductive health services \| N/A \| \| 86 \| Bergam S, Kuo C, Atujuna M, Pellowski JA, Mtukushe B, Ndevu-Qwabe N, Matiwane M, Rencken CA, Belsky M, Hoare J, Bekker LG, Harrison AD. "We Should Be Taught Self-Respect, Self-Confidence and Self-Love": Youth Perspectives of Adult Influences on Their Sexuality and Relationships Among South African Adolescents Living With HIV. Front Reprod Health. 2022 Jul 12;4:913170. doi: 10.3389/frph.2022.913170. PMID: 36303635; PMCID: PMC9580667. \| South Africa \| No \| N/A \| N/A \| \| 89 \| Demeke F, Yohannes T, Abera N, Belayneh F, Nigussie S. Youth friendly services utilization and associated factors among school youths in North Shewa Zone, Amhara Region, Ethiopia: A mixed-method study. SAGE Open Med. 2022 Aug 13;10:20503121221112025. doi: 10.1177/20503121221112025. PMID: 35993093; PMCID: PMC9382065. \| Ethiopia \| Unclear \| N/A \| N/A \| \| 119 \| Edwards PV, Roberts ST, Chelwa N, Phiri L, Nyblade L, Mulenga D, Brander C, Musheke M, Mbizvo M, Subramanian S. Perspectives of Adolescent Girls and Young Women on Optimizing Youth-Friendly HIV and Sexual and Reproductive Health Care in Zambia. Front Glob Womens Health. 2021 Oct 25;2:723620. doi: 10.3389/fgwh.2021.723620. PMID: 34816241; PMCID: PMC8594040. \| Zambia \| No \| N/A \| N/A \| \| 125 \| Madiba S, Mohlabane N. Attendance of psychosocial teen clubs and self-reported antiretroviral medication adherence: a cross section study of adolescents with perinatal HIV in the Kingdom of Lesotho. AIMS Public Health. 2021 Aug 2;8(3):541-552. doi: 10.3934/publichealth.2021044. PMID: 34395704; PMCID: PMC8334641. \| Kingdom of Lesotho \| No \| Teen clubs \| Positive \| \| 6 \| Logie CH, Okumu M, Loutet M, Berry I, Lukone SO, Kisubi N, Mwima S, Kyambadde P. Mixed-methods findings from the Ngutulu Kagwero (agents of change) participatory comic pilot study on post-rape clinical care and sexual violence prevention with refugee youth in a humanitarian setting in Uganda. Glob Public Health. 2023 Jan;18(1):2092178. doi: 10.1080/17441692.2022.2092178. Epub 2022 Jun 30. PMID: 35770702. \| Uganda \| Yes \| Comic book \| Positive \| \| 12 \| Tembo M, Simms V, Weiss HA, Bandason T, Redzo N, Larsson L, Dauya E, Nzanza T, Ishumael P, Gweshe N, Nyamwanza R, Ndlovu P, Bernays S, Chikwari CD, Mavodza CV, Renju J, Francis SC, Ferrand RA, Mackworth-Young C. High uptake of menstrual health information, products and analgesics within an integrated sexual reproductive health service for young people in Zimbabwe. Res Sq [Preprint]. 2023 Jun 26:rs.3.rs-3058045. doi: 10.21203/rs.3.rs-3058045/v1. Update in: Reprod Health. 2024 Apr 22;21(1):56. doi: 10.1186/s12978-024-01789-y. PMID: 37461550; PMCID: PMC10350165. \| Ziimibabwe \| No \| CHIEZDA \| Positive \| \| 23 \| Ayieko S, Nguku A, Kidula N. It's not just about pads! Adolescent reproductive health views in Kenya: A qualitative secondary analysis. PLOS Glob Public Health. 2023 May 22;3(5):e0001285. doi: 10.1371/journal.pgph.0001285. PMID: 37216318; PMCID: PMC10202265. \| Kenya \| No \| What Women Want campaign \| N/A \| \| 28 \| Idigbe I, Gbaja-Biamila T, Asuquo S, Nwaozuru U, Obiezu-Umeh C, Tahlil KM, Musa AZ, Oladele D, Kapogiannis B, Tucker J, Iwelunmor J, Ezechi O. Using a Designathon to Develop an HIV Self-Testing Intervention to Linkage to Care Improve Among Youths in Nigeria: Qualitative Approach Based on a Participatory Research Action Framework. JMIR Form Res. 2023 Jun 29;7:e38528. doi: 10.2196/38528. PMID: 37384385; PMCID: PMC10365579. \| Nigeria \| Yes \| Designathon \| Positive \| \| 37 \| Pleaner M, Milford C, Kutywayo A, Naidoo N, Mullick S. Sexual and reproductive health and rights knowledge, perceptions, and experiences of adolescent learners from three South African townships: qualitative findings from the Girls Achieve Power (GAP Year) Trial. Gates Open Res. 2022 Nov 30;6:60. doi: 10.12688/gatesopenres.13588.2. PMID: 37249954; PMCID: PMC10220247. \| South Africa \| No \| GAP \| Positive \| \| 250 \| Harrison L, Kumwenda M, Nyirenda L, Chilongosi R, Corbett E, Hatzold K, Johnson C, Simwinga M, Desmond N, Taegtmeyer M. "You have a self-testing method that preserves privacy so how come you cannot give us treatment that does too?" Exploring the reasoning among young people about linkage to prevention, care and treatment after HIV self-testing in Southern Malawi. BMC Infect Dis. 2022 Apr 21;22(Suppl 1):395. doi: 10.1186/s12879-022-07231-7. PMID: 35449095; PMCID: PMC902657 \| Malawi \| Yes \| HIVST \| Positive \| \| 252 \| Sidibé S, Kolié D, Grovogui FM, Kourouma K, Camara BS, Delamou A, Kouanda S. Knowledge, attitudes, and practices of health providers regarding access to and use of contraceptive methods among adolescents and youth in urban Guinea. Front Public Health. 2022 Nov 17;10:953806. doi: 10.3389/fpubh.2022.953806. PMID: 36466457; PMCID: PMC9713309. \| Guinea \| No \| Family planning services \| Both \| \| 254 \| Mkwananzi S, Baruwa OJ. Chasing the Youth Dividend in Nigeria, Malawi and South Africa: What Is the Role of Poverty in Determining the Health and Health Seeking Behaviour of Young Women? Int J Environ Res Public Health. 2022 Oct 30;19(21):14189. doi: 10.3390/ijerph192114189. PMID: 36361068; PMCID: PMC9654681. \| Nigeria, Malawi and South Africa \| No \| N/A \| N/A \| \| 261 \| Kelly J, Gittings L, Laurenzi C, Glinski CD, Mangqalaza H, Ralayo N, Langwenya N, Sidloyi L, Mbiko A, Taleni B, Saliwe B, Toska E. HIV and SRH healthcare delivery experiences of South African healthcare workers and adolescents and young people during COVID-19. Psychol Health Med. 2022 Jan-Dec;27(sup1):155-166. doi: 10.1080/13548506.2022.2108080. Epub 2022 Aug 24. PMID: 36004413. \| South Africa \| Yes \| HEY BABY \| Both \| \| 281 \| Skovdal M, Clausen CL, Magoge-Mandizvidza P, Dzamatira F, Maswera R, Nyamwanza RP, Nyamukapa C, Thomas R, Gregson S. How gender norms and 'good girl' notions prevent adolescent girls and young women from engaging with PrEP: qualitative insights from Zimbabwe. BMC Womens Health. 2022 Aug 16;22(1):344. doi: 10.1186/s12905-022-01928-2. PMID: 35974360; PMCID: PMC9379870. \| Zimbabwe \| Unclear \| PrEP \| N/A \| \| 296 \| Naledi T, Little F, Pike C, Edwards H, Robbertze D, Wagner C, London L, Bekker LG. Women of Worth: the impact of a cash plus intervention to enhance attendance and reduce sexual health risks for young women in Cape Town, South Africa. J Int AIDS Soc. 2022 Jun;25(6):e25938. doi: 10.1002/jia2.25938. PMID: 35700052; PMCID: PMC9196891. \| South Africa \| No \| Cash Transfers \| Positive \| \| 309 \| Chimatiro CS, Mpachika-Mfipa F, Tshotetsi L, Hajison PL. School-going adolescent girls' preferences and views of family planning services in Phalombe district, Malawi: A descriptive, cross-sectional study. PLoS One. 2022 May 3;17(5):e0267603. doi: 10.1371/journal.pone.0267603. PMID: 35503775; PMCID: PMC9064102. \| Malawi \| No \| Family planning services \| N/A \| \| 310 \| Zanoni B, Archary M, Sibaya T, Ramos T, Donenberg G, Shahmanesh M, Celum C, Pettifor A, Bekker LG, Haberer J. Interventions addressing the adolescent HIV continuum of care in South Africa: a systematic review and modified Delphi analysis. BMJ Open. 2022 Apr 29;12(4):e057797. doi: 10.1136/bmjopen-2021-057797. PMID: 35487726; PMCID: PMC9058810. \| South Africa \| No \| N/A \| N/A \| \| 313 \| Skovdal M, Magoge-Mandizvidza P, Dzamatira F, Maswera R, Nyamukapa C, Thomas R, Mugurungi O, Gregson S. Improving access to pre-exposure prophylaxis for adolescent girls and young women: recommendations from healthcare providers in eastern Zimbabwe. BMC Infect Dis. 2022 Apr 23;22(1):399. doi: 10.1186/s12879-022-07376-5. PMID: 35461220; PMCID: PMC9035262. \| Zimbabwe \| No \| PrEP \| N/A \| \| 433 \| Simuyaba M, Hensen B, Phiri M, Mwansa C, Mwenge L, Kabumbu M, Belemu S, Shanaube K, Schaap A, Floyd S, Fidler S, Hayes R, Ayles H, Simwinga M. Engaging young people in the design of a sexual reproductive health intervention: Lessons learnt from the Yathu Yathu ("For us, by us") formative study in Zambia. BMC Health Serv Res. 2021 Jul 29;21(1):753. doi: 10.1186/s12913-021-06696-7. PMID: 34325696; PMCID: PMC8320161. \| Zambia \| Yes \| Sexual and Reproductive Health Services  (SRHS) \| Positive \| \| 442 \| Ninsiima LR, Chiumia IK, Ndejjo R. Factors influencing access to and utilisation of youth-friendly sexual and reproductive health services in sub-Saharan Africa: a systematic review. Reprod Health. 2021 Jun 27;18(1):135. doi: 10.1186/s12978-021-01183-y. PMID: 34176511; PMCID: PMC8237506. \| SSA \| No \| SRHS \| N/A \| \| 446 \| Hailemariam S, Gutema L, Agegnehu W, Derese M. Challenges Faced by Female Out-of-School Adolescents in Accessing and Utilizing Sexual and Reproductive Health Service: A Qualitative Exploratory Study in Southwest, Ethiopia. J Prim Care Community Health. 2021 Jan-Dec;12:21501327211018936. doi: 10.1177/21501327211018936. PMID: 34032169; PMCID: PMC8155775. \| Ethiopia \| N/A \| N/A \| N/A \| \| 450 \| Abrams EA, Burke VM, Merrill KG, Frimpong C, Miti S, Mwansa JK, Denison JA. "Adolescents do not only require ARVs and adherence counseling": A qualitative investigation of health care provider experiences with an HIV youth peer mentoring program in Ndola, Zambia. PLoS One. 2021 Jun 9;16(6):e0252349. doi: 10.1371/journal.pone.0252349. PMID: 34106967; PMCID: PMC8189477. \| Zambia \| N/A \| Peer-mentoring program \| Positive \| \| 462 \| Tuhebwe, D., Babirye, S., Ssendagire, S. et al. The extent to which the design of available reproductive health interventions fit the reproductive health needs of adolescents living in urban poor settings of Kisenyi, Kampala, Uganda. BMC Public Health 21, 933 (2021). https://doi.org/10.1186/s12889-021-10933-3 \| Uganda \| No \| Reproductive health services \| N/A \| \| 463 \| Oberth G, Chinhengo T, Katsande T, Mhonde R, Hanisch D, Kasere P, Chihumela B, Madzima B. Effectiveness of the Sista2Sista programme in improving HIV and other sexual and reproductive health outcomes among vulnerable adolescent girls and young women in Zimbabwe. Afr J AIDS Res. 2021 Jul;20(2):158-164. doi: 10.2989/16085906.2021.1918733. Epub 2021 May 17. PMID: 33998958. \| Zimbabwe \| N/A \| Sista2sista \| Positive \| \| 488 \| Adams L, Crowley T. Adolescent human immunodeficiency virus self-management: Needs of adolescents in the Eastern Cape. Afr J Prim Health Care Fam Med. 2021 Feb 18;13(1):e1-e9. doi: 10.4102/phcfm.v13i1.2756. PMID: 33764137; PMCID: PMC8007991. \| South Africa \| N/A \| N/A \| N/A \| \| 544 \| Mutea L, Ontiri S, Kadiri F, Michielesen K, Gichangi P. Access to information and use of adolescent sexual reproductive health services: Qualitative exploration of barriers and facilitators in Kisumu and Kakamega, Kenya. PLoS One. 2020 Nov 12;15(11):e0241985. doi: 10.1371/journal.pone.0241985. PMID: 33180849; PMCID: PMC7660470. \| Kenya \| N/A \| N/A \| N/A \| \| 238 \| Arije, O., Hlungwani, T. & Madan, J. Key informants’ perspectives on policy- and service-level challenges and opportunities for delivering adolescent and youth-friendly health services in public health facilities in a Nigerian setting. BMC Health Serv Res 22, 1493 (2022). \| Nigeria \| N/Y \| N/Y \| N/Y \| \| 395 \| Ippoliti N, Sekamana M, Baringer L, Hope R. Using Human-Centered Design to Develop, Launch, and Evaluate a National Digital Health Platform to Improve Reproductive Health for Rwandan Youth. Glob Health Sci Pract. 2021 Nov 29;9(Suppl 2):S244-S260. doi: 10.9745/GHSP-D-21-00220. PMID: 34845048; PMCID: PMC8628501. \| Rwanda \| Yes \| Cyber Rwanda \| Positive \| \| 404 \| Odimba SO, Squires F, Ferenchick E, Mbassi SM, Chick P, Plesons M, Chandra-Mouli V. A collaborative learning approach to improving health worker performance in adolescent sexual and reproductive health service provision: a descriptive feasibility study in six health zones in the Democratic Republic of the Congo. Glob Health Action. 2021 Jan 1;14(1):1985228. doi: 10.1080/16549716.2021.1985228. PMID: 34720073; PMCID: PMC8567869. \| DRC \| Yes \| Collaborative learning approach \| Positive \| \| 411 \| Rousseau E, Katz AWK, O'Rourke S, Bekker LG, Delany-Moretlwe S, Bukusi E, Travill D, Omollo V, Morton JF, O'Malley G, Haberer JE, Heffron R, Johnson R, Celum C, Baeten JM, van der Straten A. Adolescent girls and young women's PrEP-user journey during an implementation science study in South Africa and Kenya. PLoS One. 2021 Oct 14;16(10):e0258542. doi: 10.1371/journal.pone.0258542. PMID: 34648589; PMCID: PMC8516266. \| South Africa and Kenya \| No \| PrEP \| N/A \| \| 574 \| Kidman R, Waidler J, Palermo T; Tanzania Adolescent Cash Plus Evaluation Team. Uptake of HIV testing among adolescents and associated adolescent-friendly services. BMC Health Serv Res. 2020 Sep 17;20(1):881. doi: 10.1186/s12913-020-05731-3. PMID: 32943066; PMCID: PMC7499858. \| Tanzania \| No \| HIV testing \| N/A \| \| 589 \| Jonas K, Duby Z, Maruping K, Dietrich J, Slingers N, Harries J, Kuo C, Mathews C. Perceptions of contraception services among recipients of a combination HIV-prevention interventions for adolescent girls and young women in South Africa: a qualitative study. Reprod Health. 2020 Aug 14;17(1):122. doi: 10.1186/s12978-020-00970-3. PMID: 32795366; PMCID: PMC7427945. \| South Africa \| No \| Contraception services \| N/A \| \| 592 \| Nmadu AG, Mohammed S, Usman NO. Barriers to adolescents' access and utilisation of reproductive health services in a community in north-western Nigeria: A qualitative exploratory study in primary care. Afr J Prim Health Care Fam Med. 2020 Jul 8;12(1):e1-e5. doi: 10.4102/phcfm.v12i1.2307. PMID: 32787401; PMCID: PMC7433241. \| Nigeria \| No \| N/A \| N/A \| \| 632 \| Agu, I.C., Mbachu, C.O., Okeke, C. et al. Misconceptions about transmission, symptoms and prevention of HIV/AIDS among adolescents in Ebonyi state, South-east Nigeria. BMC Res Notes 13, 244 (2020). https://doi.org/10.1186/s13104-020-05086-2 \| Nigeria \| No \| N/A \| N/A \| \| 376 \| Chingono RMS, Mackworth-Young CRS, Ross DA, Tshuma M, Chiweshe T, Nyamayaro C, Sekanevana C, Doyle AM, Weiss HA, Kohl K, Mangombe A, Madzima B, McHugh G, Ferrand RA. Designing Routine Health Checkups for Adolescents in Zimbabwe. J Adolesc Health. 2021 Dec;69(6):940-947. doi: 10.1016/j.jadohealth.2021.07.002. Epub 2021 Aug 3. PMID: 34362647; PMCID: PMC7614877. \| Zimbabwe \| Yes \| Routine health checkup \| Positive \| \| 158 \| Lavoie MC, Okui L, Blanco N, Stoebenau K, Magidson JF, Gokatweng G, Ikgopoleng K, Charurat ME, Ndwapi N. Feasibility and acceptability of peer-delivered interventions using mHealth for PrEP services among adolescent girls and young women in DREAMS program in Botswana. Glob Health Action. 2023 Dec 31;16(1):2231256. doi: 10.1080/16549716.2023.2231256. PMID: 37462118; PMCID: PMC10355684. \| Botswana \| No \| mHealth \| Positive \| \| 963 \| Self, A., Chipokosa, S., Misomali, A. et al. Youth accessing reproductive health services in Malawi: drivers, barriers, and suggestions from the perspectives of youth and parents. Reprod Health 15, 108 (2018). https://doi.org/10.1186/s12978-018-0549-9 \| Malawi \| No \| N/A \| N/A \| \| 969 \| McCarraher DR, Packer C, Mercer S, Dennis A, Banda H, Nyambe N, Stalter RM, Mwansa JK, Katayamoyo P, Denison JA. Adolescents living with HIV in the Copperbelt Province of Zambia: Their reproductive health needs and experiences. PLoS One. 2018 Jun 5;13(6):e0197853. doi: 10.1371/journal.pone.0197853. PMID: 29870562; PMCID: PMC5988282. \| Zambia \| No \| N/A \| N/A \| \| 988 \| Pilgrim N, Jani N, Mathur S, Kahabuka C, Saria V, Makyao N, Apicella L, Pulerwitz J. Provider perspectives on PrEP for adolescent girls and young women in Tanzania: The role of provider biases and quality of care. PLoS One. 2018 Apr 27;13(4):e0196280. doi: 10.1371/journal.pone.0196280. PMID: 29702659; PMCID: PMC5922529. \| Tanzania \| No \| PrEP \| N/A \| \| 1080 \| Kujawski SA, Freedman LP, Ramsey K, Mbaruku G, Mbuyita S, Moyo W, Kruk ME. Community and health system intervention to reduce disrespect and abuse during childbirth in Tanga Region, Tanzania: A comparative before-and-after study. PLoS Med. 2017 Jul 11;14(7):e1002341. doi: 10.1371/journal.pmed.1002341. PMID: 28700587; PMCID: PM \| Tanzania \| Yes \| Staha \| Positive \| \| 682 \| Maseko B, Hill LM, Phanga T, Bhushan N, Vansia D, Kamtsendero L, Pettifor AE, Bekker LG, Hosseinipour MC, Rosenberg NE. Perceptions of and interest in HIV pre-exposure prophylaxis use among adolescent girls and young women in Lilongwe, Malawi. PLoS One. 2020 Jan 13;15(1):e0226062. doi: 10.1371/journal.pone.0226062. PMID: 31929547; PMCID: PMC6957134. \| Malawi \| No \| PrEP \| Positive \| \| 725 \| Chipokosa S, Pattnaik A; NEP Malawi Technical Task Team*; Misomali A, Mohan D, Peters M, Kachale F, Ndawala J, Marx MA. How strong are Malawi's family planning programs for adolescent and adult women? Results of a national assessment of implementation strength conducted by Malawi's National Evaluation Platform. J Glob Health. 2019 Dec;9(2):020901. doi: 10.7189/jogh.09.020901. PMID: 33282227; PMCID: PMC7689283. \| Malawi \| No \| Family planning services \| N/A \| \| 804 \| Jonas K, Roman N, Reddy P, Krumeich A, van den Borne B, Crutzen R. Nurses' perceptions of adolescents accessing and utilizing sexual and reproductive healthcare services in Cape Town, South Africa: A qualitative study. Int J Nurs Stud. 2019 Sep;97:84-93. doi: 10.1016/j.ijnurstu.2019.05.008. Epub 2019 May 22. PMID: 31200221. \| South Africa \| No \| SRHS \| N/A \| \| 895 \| Chanda BC, Likwa RN, Zgambo J, Tembo L, Jacobs C. Acceptability of option B+ among HIV positive women receiving antenatal and postnatal care services in selected health centre's in Lusaka. BMC Pregnancy Childbirth. 2018 Dec 29;18(1):510. doi: 10.1186/s12884-018-2142-1. PMID: 30594161; PMCID: PMC6311017. \| Zambia \| No \| ART \| Positive \| \| 903 \| Mochache V, Irungu E, El-Busaidy H, Temmerman M, Gichangi P. "Our voices matter": a before-after assessment of the effect of a community-participatory intervention to promote uptake of maternal and child health services in Kwale, Kenya. BMC Health Serv Res. 2018 Dec 4;18(1):938. doi: 10.1186/s12913-018-3739-9. PMID: 30514292; PMCID: PMC6280535. \| Kenya \| Yes \| Dialogue Model \| Positive \| \| 1108 \| Ndongmo TN, Ndongmo CB, Michelo C. Sexual and reproductive health knowledge and behavior among adolescents living with HIV in Zambia: a case study. Pan Afr Med J. 2017 Feb 20;26:71. doi: 10.11604/pamj.2017.26.71.11312. PMID: 28451048; PMCID: PMC5398862. \| Zambia \| Unclear \| N/A \| N/A \| \| 1136 \| Renzaho AM, Kamara JK, Georgeou N, Kamanga G. Sexual, Reproductive Health Needs, and Rights of Young People in Slum Areas of Kampala, Uganda: A Cross Sectional Study. PLoS One. 2017 Jan 20;12(1):e0169721. doi: 10.1371/journal.pone.0169721. PMID: 28107371; PMCID: PMC5249247. \| Uganda \| No \| N/A \| N/A \| \| 1384 \| Aninanya GA, Debpuur CY, Awine T, Williams JE, Hodgson A, Howard N. Effects of an adolescent sexual and reproductive health intervention on health service usage by young people in northern Ghana: a community-randomised trial. PLoS One. 2015 Apr 30;10(4):e0125267. doi: 10.1371/journal.pone.0125267. PMID: 25928562; PMCID: PMC4415997. \| Ghana \| No \| Social learning intervention \| Positive \| \| 1403 \| Dellar RC, Dlamini S, Karim QA. Adolescent girls and young women: key populations for HIV epidemic control. J Int AIDS Soc. 2015 Feb 26;18(2 Suppl 1):19408. doi: 10.7448/IAS.18.2.19408. PMID: 25724504; PMCID: PMC4344544. \| South Africa \| No \| N/A \| N/A \| \| 1329 \| Hoopes AJ, Chandra-Mouli V, Steyn P, Shilubane T, Pleaner M. An Analysis of Adolescent Content in South Africa's Contraception Policy Using a Human Rights Framework. J Adolesc Health. 2015 Dec;57(6):617-23. doi: 10.1016/j.jadohealth.2015.08.012. PMID: 26592330; PMCID: PMC5357766. \| South Africa \| No \| N/A \| N/A \| \| 3300 \| Kasibante, P., Kiboss, J. K., Atuhairwe, C., Taremwa, I. M. (2020). Individual, health facility-related, and psychosocial determinants of retention in chronic HIV care among HIV-positive young people in Mukono Municipality, Uganda. HIV & AIDS Review, 19(2), 99-105. https://doi.org/10.5114/hivar.2020.96386 \| Uganda \| No \| ART \| N/A \| \| 3523 \| Hodes R, Doubt J, Toska E, Vale B, Zungu N, Cluver L. The stuff that dreams are made of: HIV-positive adolescents' aspirations for development. J Int AIDS Soc. 2018 Feb;21 Suppl 1(Suppl Suppl 1):e25057. doi: 10.1002/jia2.25057. PMID: 29485764; PMCID: PMC5978641. \| South Africa \| Yes \| DREAM Clinics \| Positive \| \| 3796 \| Taaffe J, Cheikh N, Wilson D. The use of cash transfers for HIV prevention--are we there yet? Afr J AIDS Res. 2016;15(1):17-25. doi: 10.2989/16085906.2015.1135296. PMID: 27002355. \| Africa \| No \| Cash transfers \| Positive \| \| 4843 \| Ngabirano TD, Saftner MA, McMorris BJ. Exploring Health Behaviors in Ugandan Adolescents Living in Rural Fishing Communities. J Sch Nurs. 2022 Apr;38(2):148-160. doi: 10.1177/1059840520947142. Epub 2020 Aug 6. PMID: 32757810. \| Uganda \| No \| N/A \| N/A \| \| 4863 \| Jerene D, Tiberg I, Hallström I. How Can Clinical Outcomes among Adolescents Living with HIV in Ethiopia be Improved? Healthcare Professionals' Perspectives. Compr Child Adolesc Nurs. 2021 May 7;45(2):191-200. doi: 10.1080/24694193.2021.1914774. PMID: 33961528.. \| Ethiopia \| No \| N/A \| N/A \| \| 2966 \| Somefun OD, Casale M, Haupt Ronnie G, Desmond C, Cluver L, Sherr L. Decade of research into the acceptability of interventions aimed at improving adolescent and youth health and social outcomes in Africa: a systematic review and evidence map. BMJ Open. 2021 Dec 20;11(12):e055160. doi: 10.1136/bmjopen-2021-055160. PMID: 34930743; PMCID: PMC8689197. \| Africa \| No \| N/A \| N/A \| \| 3117 \| Kyegombe N, Zuma T, Hlongwane S, Nhlenyama M, Chimbindi N, Birdthistle I, Floyd S, Seeley J, Shahmanesh M. A qualitative exploration of the salience of MTV-Shuga, an edutainment programme, and adolescents' engagement with sexual and reproductive health information in rural KwaZulu-Natal, South Africa. Sex Reprod Health Matters. 2022 Dec;30(1):2083809. doi: 10.1080/26410397.2022.2083809. PMID: 35929964; PMCID: PMC9448405. \| South Africa \| Yes \| MTV Shuga \| Positive \| \| 3565 \| Iwelunmor J, Blackstone S, Nwaozuru U, Conserve D, Iwelunmor P, Ehiri JE. Sexual and reproductive health priorities of adolescent girls in Lagos, Nigeria: findings from free-listing interviews. Int J Adolesc Med Health. 2017 May 17;30(5):/j/ijamh.2018.30.issue-5/ijamh-2016-0105/ijamh-2016-0105.xml. doi: 10.1515/ijamh-2016-0105. PMID: 28525320. \| Nigeria \| No \| N/A \| N/A \| \| 3792 \| Klinger A, Asgary R. Implementation and evaluation of a curriculum to teach reproductive health to adolescents in northern Madagascar. Int Health. 2016 May;8(3):179-86. doi: 10.1093/inthealth/ihv057. Epub 2015 Sep 7. PMID: 26346692. \| Madagascar \| No \| School Curriculum on HIV/STIs and family planning \| Positive \| \| 4938 \| Nmadu, A. G., Mohamed, S., & Usman, N. O. (2020). Adolescents’ utilization of reproductive health services in Kaduna, Nigeria: the role of stigma. Vulnerable Children and Youth Studies, 15(3), 246–256. https://doi.org/10.1080/17450128.2020.1800156 \| Nigeria \| No \| N/A \| N/A \| \| 5344 \| Gardsbane, Diane & Bukuluki, Paul. (2023). Keeping the Essentials in Place: Lessons Learned from a Qualitative Study of DREAMS in Northern Uganda. Adolescents. 3. 290-304. 10.3390/adolescents3020021. \| Uganda \| No \| DREAMS \| Positive \| \| 5619 \| Gibbs A, Willan S, Jama-Shai N, Washington L, Jewkes R. 'Eh! I felt I was sabotaged!': facilitators' understandings of success in a participatory HIV and IPV prevention intervention in urban South Africa. Health Educ Res. 2015 Dec;30(6):985-95. doi: 10.1093/her/cyv059. PMID: 26590246; PMCID: PMC4654179. \| South Africa \| Yes \| Stepping Stones and Creating Futures \| Positive \| \| 3681 \| Burke E, Kébé F, Flink I, van Reeuwijk M, le May A. A qualitative study to explore the barriers and enablers for young people with disabilities to access sexual and reproductive health services in Senegal. Reprod Health Matters. 2017 May;25(50):43-54. doi: 10.1080/09688080.2017.1329607. Epub 2017 May 30. PMID: 28784062. \| Senegal \| No \| SRHS \| N/A \| \| 1723 \| Nsakala, G.V., Coppieters, Y. & Kayembe, P.K. An innovative approach to using both cellphones and the radio to identify young people’s sexual concerns in Kinshasa, Democratic Republic of Congo. Arch Public Health 72, 21 (2014). https://doi.org/10.1186/2049-3258-72-21. \| DRC \| Yes \| "Please Doctor" Radio program \| Positive \| \| 1174 \| Hall KS, Manu A, Morte E, Dalton VK, Challa S, Loll D, Dozier JL, Zochowski MK, Boakye A, Harris LH. Bad girl and unmet family planning need among Sub-Saharan African adolescents: the role of sexual and reproductive health stigma. Qual Res Med Healthc. 2018 May 30;2(1):55-64. doi: 10.4081/qrmh.2018.7062. PMID: 30556052; PMCID: PMC6292434. \| Ghana \| No \| SRHS \| N/A \| \| 1179 \| Ogu R, Maduka O, Alamina F, Adebiyi O, Agala V, Eke G, Porbeni I, Offor N, Abam C, Nte A, Okonofua F. Mainstreaming youth-friendly health services into existing primary health care facilities: experiences from South-South Nigeria. Int J Adolesc Med Health. 2018 Jan 25;32(3). doi: 10.1515/ijamh-2017-0151. PMID: 29369812. \| Nigeria \| No \| YFHS \| Positive \| \| 1186 \| Kuyinu YA, Femi-Adebayo TT, Odugbemi BA, Ukatu EE. Causative factors for sexual and reproductive health status of pregnant adolescent girls in urban communities of Lagos, Nigeria. Int J Adolesc Med Health. 2017 Sep 15;32(2):/j/ijamh.2020.32.issue-2/ijamh-2017-0104/ijamh-2017-0104.xml. doi: 10.1515/ijamh-2017-0104. PMID: 28915113. \| Nigeria \| No \| N/A \| N/A \| |
| --- | --- | --- | --- | --- | --- | --- | --- | --- | --- | --- | --- | --- | --- | --- | --- | --- | --- | --- | --- | --- | --- | --- | --- | --- | --- | --- | --- | --- | --- | --- | --- | --- | --- | --- | --- | --- | --- | --- | --- | --- | --- | --- | --- | --- | --- | --- | --- | --- | --- | --- | --- | --- | --- | --- | --- | --- | --- | --- | --- | --- | --- | --- | --- | --- | --- | --- | --- | --- | --- | --- | --- | --- | --- | --- | --- | --- | --- | --- | --- | --- | --- | --- | --- | --- | --- | --- | --- | --- | --- | --- | --- | --- | --- | --- | --- | --- | --- | --- | --- | --- | --- | --- | --- | --- | --- | --- | --- | --- | --- | --- | --- | --- | --- | --- | --- | --- | --- | --- | --- | --- | --- | --- | --- | --- | --- | --- | --- | --- | --- | --- | --- | --- | --- | --- | --- | --- | --- | --- | --- | --- | --- | --- | --- | --- | --- | --- | --- | --- | --- | --- | --- | --- | --- | --- | --- | --- | --- | --- | --- | --- | --- | --- | --- | --- | --- | --- | --- | --- | --- | --- | --- | --- | --- | --- | --- | --- | --- | --- | --- | --- | --- | --- | --- | --- | --- | --- | --- | --- | --- | --- | --- | --- | --- | --- | --- | --- | --- | --- | --- | --- | --- | --- | --- | --- | --- | --- | --- | --- | --- | --- | --- | --- | --- | --- | --- | --- | --- | --- | --- | --- | --- | --- | --- | --- | --- | --- | --- | --- | --- | --- | --- | --- | --- | --- | --- | --- | --- | --- | --- | --- | --- | --- | --- | --- | --- | --- | --- | --- | --- | --- | --- | --- | --- | --- | --- | --- | --- | --- | --- | --- | --- | --- | --- | --- | --- | --- | --- | --- | --- | --- | --- | --- | --- | --- | --- | --- | --- | --- | --- | --- | --- | --- | --- | --- | --- | --- | --- | --- | --- | --- | --- | --- | --- | --- | --- | --- | --- | --- | --- | --- | --- | --- | --- | --- | --- | --- | --- | --- | --- | --- | --- | --- | --- | --- | --- | --- | --- | --- | --- | --- | --- | --- | --- | --- | --- | --- | --- | --- | --- | --- | --- | --- | --- | --- | --- | --- | --- | --- | --- | --- | --- | --- | --- | --- | --- | --- | --- | --- | --- | --- | --- | --- | --- | --- | --- | --- | --- | --- | --- | --- | --- | --- | --- | --- | --- | --- | --- | --- | --- | --- | --- | --- | --- | --- | --- | --- | --- | --- | --- | --- | --- | --- | --- | --- | --- | --- | --- | --- | --- | --- | --- | --- | --- | --- | --- | --- | --- | --- | --- | --- | --- | --- | --- | --- | --- | --- | --- | --- | --- | --- | --- | --- | --- | --- | --- | --- | --- | --- | --- | --- | --- | --- | --- | --- | --- | --- | --- | --- | --- | --- | --- | --- | --- | --- | --- | --- | --- | --- | --- | --- | --- | --- | --- | --- | --- | --- | --- | --- | --- | --- | --- | --- | --- | --- | --- | --- | --- | --- | --- | --- | --- | --- |
