# Supplementary material for: A systematic review of the effectiveness of participatory, health system-based interventions to improve the sexual and reproductive health and rights of adolescent girls and young women in Sub-Saharan Africa
Source: Sex Reprod Health Matters. 2026 Mar 18;33(1):2643037. doi: 10.1080/26410397.2026.2643037 (PMC13103997; doi:10.1080/26410397.2026.2643037)
Supplement: Supplemental Table 2. Quality assessments of four included studies. [file ZRHM_A_2643037_SM3930.docx]

SUPPLEMENTAL TABLE 2: Quality Assessment of Included Articles

Scoring – Good =4, Fair =3, Poor =2 (Possible total of 32)

1. Article#439 - 30.5 points (Stangl et al., Tikambusane)
2. Article#474 - 29 points (Manda et al., Girls-Only Clubs)
3. Article #172 - 28.5 points (Nakalega et al, HIVST and PReP)
4. Article#375 & #4613 - 26.5 points (Mavodza et al., testing preferences)

| **ARTICLE SECTION** | **ARTICLE RATING** | | | | **GOOD** | **FAIR** | **POOR** |
| --- | --- | --- | --- | --- | --- | --- | --- |
|  | **#1** | **#2** | **#3** | **#4** |  |  |  |
| 1 Intro/Objectives:  Is background provided Is there a clear statement of research objectives? | 3.5 | 4 | 4 | 4 | -Full but concise background.  -Up-to-date literature review highlighting gaps.  -Clear statement of objectives including research questions. | -Some background & literature review.  -Research questions outlined. | -Some background but no objectives/questions, OR  -Objectives given but inadequate background. |
| 2 Methods/Data:  Is the methodology appropriate & clearly explained? | 3.5 | 3.5 | 4 | 3.5 | Methods appropriate & clearly described (e.g., questionnaires  included).  -Qualifications of research team discussed.  --Details of data collection & recording provided. | -Methods appropriate, but no description of guiding questions, only that they told “stories”. | -Questionable if method is appropriate.  -Method described inadequately.  -Little data description. |
| 3 Sampling:  Sampling strategy appropriate to meet objectives? | 4 | 4 | 4 | 2.25 | -Details provided on sample population (age/sex/race/context) & how recruited.  -Why group(s) selected. Does it mention if study population is part of larger group.  -Sample size justified & appropriate for study.  -Response rates shown & explained. | -Sample size justified.  -Most necessary information given, but some missing. | -Sampling mentioned but few descriptive details. |
| 4 Data Analysis: | 4 | 4 | 4 | 4 | -Clear description of how analysis done, e.g.  Qualitative studies:  -Description of how themes derived/ respondent validation or triangulation.  Quantitative studies:  -Hypothesis driven.  -Calculations correct/statistical significance discussed. | Qualitative:  -Descriptive discussion of analysis.  Quantitative  -Methods incompletely described | -Minimal details about analysis. |
| 5 Ethics & Bias:  Ethical issues addressed &necessary ethical approval obtained? Researcher- participant relationships considered? | 4 | 4 | 3.5 | 3.5 | Ethics:  -Where necessary, issues of confidentiality, sensitivity, &  consent addressed.  Bias:  -Researcher reflective &/or aware of own bias. | Ethics/bias issues  acknowledged but insufficiently addressed. | -Brief mention of ethical and bias issues only. |
| 6 Findings/  Results:  Are the findings clearly presented? | 4 | 4 | 3.5 | 3.5 | -Findings explicit, easy to understand, in logical progression.  -Importance  /effectiveness of participation discussed & how measured?  -Results of any health workers participating & implications discussed?  -Tables, if present, explained in text.  -Results relate directly to objectives.  -Sufficient data presented to support findings. | -Findings incompletely explained.  -Data presented relate directly to results. | -Findings presented haphazardly, not explained in detail. |
| 7 Transferability/  Generalizability:  Are findings transferable (generalizable) to a wider population or potentially other settings? Are they related to broader research context? | 3.5 | 2.5 | 2.5 | **2.75** | -Study context & setting described sufficiently to permit comparison with other contexts, PLUS high score on Item 3 (sampling). | -Some context & setting described, but more needed to replicate/compare with other studies PLUS fair score or higher in Item 3. | -Minimal description of context/setting. |
| 8 Implications &  Usefulness:  Importance of findings to policy &/or practice? | 4 | 3 | 3 | 3 | (1) Demonstrates that paper contributes new  understanding, insight, perspective.  (2) Suggests ideas for further research.  (3) Suggests implications for policy &/or practice. | -2 of the 3 elements in column 3 included and convincing. | -Only 1 of 3 elements in column 3 included and convincing. |

*Slightly adapted) from Hawker et al. (2002)
